# Divergent therapeutic and prognostic impacts of immunogenic features in undifferentiated pleomorphic sarcoma and myxofibrosarcoma

**DOI:** 10.1007/s00262-025-04123-y

**Published:** 2025-07-02

**Authors:** Siddh van Oost, Debora M. Meijer, Zeynep B. Erdem, Marieke E. IJsselsteijn, Jessica Roelands, Suk Wai Lam, Melissa S. Boejharat, Brendy E. W. M. van den Akker, Ruud van der Breggen, Inge H. Briare-de Bruijn, Lukas J. A. C. Hawinkels, Anouk A. Kruiswijk, Manon van der Ploeg, Pauline M. Wijers-Koster, Rick L. Haas, Michiel A. J. van den Sande, Noel F. C. C. de Miranda, Judith V. M. G. Bovee

**Affiliations:** 1 Department of Pathology, Leiden University Medical Center, Leiden, The Netherlands; 2 Leiden Center for Computational Oncology, LUMC, Leiden, The Netherlands; 3 Department of Gastroenterology and Hepatology, Leiden University Medical Center, Leiden, The Netherlands; 4 Department of Orthopedic Surgery, Leiden University Medical Center, Leiden, The Netherlands; 5 Department of Radiotherapy, The Netherlands Cancer Institute, Amsterdam, The Netherlands; 6 Department of Radiotherapy, Leiden University Medical Center, Leiden, The Netherlands

**Keywords:** Undifferentiated pleomorphic sarcomas, Myxofibrosarcomas, Immune microenvironment, Metastasis-free survival, Radiotherapy

## Abstract

Undifferentiated pleomorphic sarcoma (UPS) and myxofibrosarcoma (MFS) are genetically complex soft tissue sarcomas with distinct morphological features. Treatment typically involves surgery, often combined with neoadjuvant chemo- or radiotherapy. To better understand the immunobiology of these sarcomas and its associations with treatment response and prognosis, we performed transcriptomic and immunophenotypic profiling. RNA sequencing was performed on 13 UPS and 10 MFS, and immunological profiles were compared with soft tissue sarcoma data from The Cancer Genome Atlas (*n* = 206 including 44 UPS and 17 MFS). Immune contextures were further evaluated in 14 UPS and 15 MFS using imaging mass cytometry. Characterization of T cell and macrophage infiltration in tumors was further assessed in 23 UPS and 22 MFS through multispectral immunofluorescence and immunohistochemical analysis. UPS and MFS demonstrated immunogenic features compared to other soft tissue sarcomas, with subsets of UPS and MFS demonstrating high T cell infiltration, while UPS demonstrated a higher infiltration by myeloid cells as compared to MFS. Prognostically, T cells and CD68^+^CD163^+^ macrophages were associated with metastasis-free survival in UPS but not in MFS. Notably, in UPS, neoadjuvant radiotherapy appeared to induce cytotoxic T cell infiltration and depletion of myeloid cells, whereas these effects were not observed in MFS. These findings highlight important differences in the immunobiology of UPS and MFS with therapeutic and prognostic implications. These differences should be taken into account given the growing availability of immunotherapeutic options for treating patients with soft tissue sarcomas.

**Supplementary Information:**

The online version contains supplementary material available at 10.1007/s00262-025-04123-y.

## Introduction

Soft tissue sarcomas (STSs) are a rare and diverse group of malignant mesenchymal neoplasms that predominantly arise in the extremities or trunk. Undifferentiated pleomorphic sarcoma (UPS) and myxofibrosarcoma (MFS) are two subtypes distinguished by their morphological features and lack of specific line of differentiation. UPS (also known as undifferentiated soft tissue sarcoma) present exclusively as intermediate- or high-grade deep-seated tumors, while MFS encompass a spectrum of low- to high-grade tumors and can occur either superficially or deep-seated [1]. Localized disease is typically treated with surgery, often complemented by (neo-) adjuvant radiotherapy depending on tumor size, grade, and anatomical location [2]. However, clinical outcomes remain poor, with approximately 15–45% of patients experiencing local recurrence or distant metastases [3–5]. As options for systemic therapy in recurrent disease are limited, improving clinical management through the development of novel therapeutic approaches is crucial.

Genomically, UPS and MFS present complex and heterogeneous profiles characterized by extensive chromosomal alterations and few recurrent mutation targets (e.g., *TP53*, *RB1, CDKN2A, ATRX*) [6, 7]. Additionally, these tumors display similar transcriptional and epigenetic profiles when compared to other STS [6–9]. Despite these overlaps, their clinical behavior differs considerably: UPS is more prone to metastasize, with distinct responses to neoadjuvant therapy, whereas MFS show a higher propensity for local recurrence, suggesting underlying biological differences [3, 10]. The contribution of the immune microenvironment to these differing outcomes remains unclear.

To address this, we characterized the immune microenvironment of UPS and MFS using transcriptomic and immunophenotypic profiling. Immune-related transcriptional profiles were compared with STS from The Cancer Genome Atlas (TCGA), and immune cell populations were associated with metastasis-free and disease-specific survival. Moreover, the effect of radiotherapy on the immune microenvironment was explored. Our study underscores distinct immunobiological features between UPS and MFS, with potential implications for developing tailored therapeutic strategies.

## Methods

### Patient samples

Formalin-fixed paraffin-embedded (FFPE) and snap-frozen material was collected for 16 UPS and 17 MFS patients diagnosed between 2008 and 2021. This discovery cohort comprised high- and low-grade MFS, with patients receiving surgery alone, post-operative radiotherapy or pre-operative radiotherapy. Subsequently, the cohort was expanded to include FFPE biopsy samples from an additional 14 UPS and 16 MFS patients diagnosed between 2011 and 2023. These cases were exclusively high-grade tumors that underwent neoadjuvant radiotherapy.

For imaging mass cytometry (IMC), regions representative of the tumor’s immune microenvironment were identified on hematoxylin and eosin (H&E)-stained sections of FFPE tissue by a soft tissue tumor pathologist (JVMGB). Tissue microarrays (TMAs) were then constructed using a TMA Master (3DHISTECH). Each resection contributed four cores (1.6 mm in diameter), with two central and two peripheral cores, while biopsies provided two cores. For immunohistochemistry (IHC)/immunofluorescence (IF), FFPE blocks of biopsies with sufficient tumor tissue were selected for whole slide imaging. Pathological response to radiotherapy was assessed by two soft tissue tumor pathologists (JVMGB and SWL), who defined the modified EORTC response score including percentage of necrosis, hyalinization/fibrosis and viable tumor adding up to 100% of tumor volume [11, 12].

### RNA sequencing

RNA sample processing and sequencing were performed as previously described on treatment-naïve tumors from 13 UPS and 10 MFS [13]. In short, RNA was extracted from snap-frozen tissues using TRIzol and isopropanol/ethanol, followed by purification with the RNeasy kit, including DNase treatment. Paired-end 150 base-pair (bp) reads were generated on a NovaSeq6000 Illumina at GenomeScan (Leiden, The Netherlands). Sequencing data were processed with the RNAseq BioWDL pipeline from the Sequencing Analysis Support Core (https://biowdl.github.io/, LUMC, The Netherlands). Reads were aligned to the hg38 reference genome using STAR, and gene expression was quantified using HTSeq-count [14]. The processed data were stored as a matrix containing gene counts per sample.

### Integration with TCGA-STS dataset

RNA sequencing (RNAseq) data from 206 revised cases of primary STS from TCGA [6] were downloaded and processed using TCGA Assembler (R, v.2.0.3). This dataset included UPS (*n* = 44), MFS (*n* = 17; including 3 low-grade), dedifferentiated liposarcomas (*n* = 50), soft tissue leiomyosarcomas (*n* = 53; including 11 low-grade), uterine leiomyosarcomas (*n* = 27; including 1 low-grade), malignant peripheral nerve sheath tumors (*n* = 5), and synovial sarcomas (*n* = 10). Gene symbols were converted to HGNC gene symbols and Entrez Gene identifiers using biomaRt (R, v.2.60). The dataset was filtered for overlapping genes with our own RNAseq dataset, referred to as the Leiden Center for Computational Oncology (LCCO) dataset, using Entrez Gene identifiers. Since TCGA data were in transcripts per million (TPM), the processed RNAseq counts from the LCCO cohort were converted to TPM with IOBR (R, v.0.99.8), which also removed genes without HGNC symbols. The LCCO and TCGA datasets were then merged based on HGNC symbols. Normalization was performed within lanes and between lanes by using EDASeq (R, v.2.38), after which the data were quantile-normalized with preprocessCore (v.1.66.0) and log2-transformed.

### Gene expression analysis

Batch correction was performed with limma (R, v.3.60.0) to compare immune-related gene expression between our dataset and TCGA dataset. As described previously, the immunologic constant of rejection (ICR) gene signature was utilized to characterize the Th1-like inflammatory state of the STS microenvironment [15, 16]. Additionally, immunological composition was evaluated with MCPcounter (R, v.1.2.0), which clusters samples into previously established Sarcoma Immune Classes (SIC) by using TCGA-STS data [17]. Z-scores were calculated per gene/cell population to visualize the immune-related transcriptional signatures using ComplexHeatmap (R, v.2.20.0). The attributed clusters were visualized per subtype, per dataset in bar plots with ggplot2 (R, v.3.5.1).

### Imaging mass cytometry

The conjugation of BSA-free antibodies and immunodetection using a 40-marker panel that provides an overall description of the immune cell composition (Supplementary Table 1) were carried out as previously described [13, 18]. Briefly, four-micrometer sections of the generated TMAs were deparaffinized, rehydrated and subjected to heat-induced antigen retrieval in a low pH antigen retrieval solution (pH 6, Thermo Fisher Scientific). The first set of primary antibodies was incubated overnight at 4 °C. Following washes with PBS supplemented with 1% BSA and 0.05% Tween, sections were incubated with conjugated anti-mouse (Abcam, ab6708) and anti-rabbit (Abcam, ab6701) antibodies for one hour at room temperature. Next, the second batch of primary antibodies was applied for five hours at room temperature, followed by an overnight incubation at 4 °C with the final antibody batch. Finally, DNA was stained using 1.25 µM Cell-ID™ Intercalator-Ir (Standard BioTools) for five minutes. ROIs of 1000 × 1000 µm were ablated with a Hyperion mass cytometry imaging system (Standard BioTools) at the Flow Core Facility (LUMC, Leiden). Imaging data were acquired with CyTOF Software (v.7.0) and exported using MCD Viewer (v.1.0.5).

### Imaging mass cytometry analysis

Analysis of the IMC data was performed as described previously [13, 19]. In short, images were normalized in MATLAB (v.R2021a) and binarized in Ilastik (v.1.3.3). Probability masks were generated in Ilastik and used for cell segmentation in CellProfiler (v.2.2.0). Single-cell FCS files were generated in ImaCyte [20] and utilized for phenotyping in CytoSplore (v.2.3.1). Mean cell density per mm^2^ was calculated for every sample. Lineage markers used for phenotyping are shown in Supplementary Table 2. The relative abundance of phenotypes was calculated using the total number of cells per sample and visualized using ComplexHeatmap (R, v.2.20.0).

### Multispectral immunofluorescent imaging

A T cell Opal panel comprising four markers was developed for IF imaging of pre-treatment biopsies using a Vectra system (Akoya Biosciences) as described previously [13, 21]. The panel included anti-PD-1 (D4W2J, 1:250, Cell Signaling Technology, #86,163), anti-CD8α (D8A8Y, 1:500, Cell Signaling Technology, #85,336), anti-CD3ε (EP449E, 1:1000, Abcam, ab52959) and anti-CD4 (EPR6855, 1:2000, Abcam, ab133616) antibodies. Each antibody was paired with a specific opal fluorophore in an optimized staining sequence to maximize intensity and specificity: CD8-Opal 690 (1:100), CD4-Opal 570 (1:400), PD-1-Opal 620 (1:100), and CD3-Opal 520 (1:400). Endogenous peroxidase was blocked with 0.3% hydrogen peroxide in methanol, antigen retrieval was performed with citrate buffer (pH 6.0) and tissue was blocked with SuperBlock™ Blocking Buffer (Thermo Fisher Scientific). Slides were incubated with a primary antibody for one hour at room temperature, washed with PBS-Tween (0.05%) and incubated with BrightVision DPVO-HRP (Immunologic) for 10 min. After washing, the first Opal reagent (Akoya Bioscience), diluted in Opal amplification diluent, was applied for 10 min. Slides were then heated in citrate buffer (pH 6.0) for 15 min at a reduced wattage and washed with PBS-Tween. This process was repeated for the remaining antibodies. Finally, slides were incubated with DAPI (1:1000) for five minutes and mounted with ProLong™ Gold Antifade Mountant (Thermo Fisher Scientific). Up to five ROIs per slide were selected based on consecutive hematoxylin-and-eosin-stained slides and imaged at 20 × magnification. Image processing was performed using inForm (v.2.4) and analyzed with QuPath (v.0.3.1). Due to nonspecific staining, CD4 was excluded from the analysis. T cells (CD3^+^) were classified as CD8^+^ or CD8^−^ and further categorized based on PD-1 expression (PD-1^+^ or PD-1^−^). Cell counts per image (1.5 mm × 2 mm) were normalized per patient as counts per mm^2^ tissue.

### Double immunohistochemical detection

Double IHC was performed to identify macrophages in pre-treatment biopsies using the ImmPRESS Duet Double Staining Polymer Kit (HRP Anti-Mouse IgG-brown, AP Anti-Rabbit IgG-magenta, Vector Laboratories, MP-7724-15). Anti-CD163 (10D6, Mouse, 1:400, MONOSAN, MONX10445) and anti-CD68 (D4B9C, Rabbit, 1:3200, Cell Signaling Technology, #76,437) antibodies were applied on the same slide following the manufacturer’s protocol. Slides were scanned using a Pannoramic™ 480 (3D HISTECH) slide scanner and analyzed with QuPath. Macrophages were identified using the optical density sum for cell detection, thereby excluding most CD68^+^ and/or CD163^+^ tumor cells. The same ROIs were selected as for the T cell imaging, and macrophage counts per ROI were normalized to counts per mm^2^ tissue. A detailed description of the used workflow in QuPath is presented as Supplementary Methods.

### Statistical analyses

To associate the immune microenvironment with clinical outcome in the validation cohort, patients were stratified as T cell high or T cell low based on the median total T cell count per sarcoma subtype. The same was done based on the CD68^+^CD163^−^ macrophage density and CD68^+^CD163^+^ macrophage density. To correlate T cell infiltration with CD68^+^CD163^+^ macrophage infiltration, a heatmap was generated using the mean cell densities per mm^2^ from the IF and double IHC analyses. Furthermore, the T cell-to-macrophage ratio was calculated by dividing the total T cell density by the CD68^+^CD163^+^ macrophage density per sample, as identified by IF and double IHC, respectively. For survival analysis, patients were stratified as high or low based on the median T cell-to-macrophage ratio.

Kaplan–Meier survival curves were generated using the survminer (R, v.0.4.9) and survival (R, v3.6–4) packages in R. Disease-specific survival was defined as the time until death due to the disease. Given the high number of censored cases in the cohort, statistical significance was assessed using the log-rank *P* value. Univariate and multivariate Cox proportional hazards analyses were conducted in R, with only variables found significant in the univariate analysis included in the multivariate model.

## Results

### Clinicopathological characteristics are prognostic in MFS, but not in UPS

In this study, a total of 63 patients were enrolled, including 30 individuals diagnosed with UPS and 33 with MFS. Initially, we performed RNAseq and/or IMC on treatment-naïve samples from 16 UPS (all pre-surgical biopsies) and 17 MFS (comprising 9 pre-surgical biopsies and 8 resections) to characterize the immunological landscape of these tumors. Findings were validated using IF and IHC in an extended cohort of treatment-naïve cases, consisting of 23 UPS and 22 high-grade MFS (all pre-surgical biopsies), which partly overlapped with the discovery cohort (9 UPS and 6 MFS), and were also used to investigate associations with clinical outcomes. In addition, to assess the impact of neoadjuvant radiotherapy, we analyzed surgical resection specimens from 13 UPS and 8 MFS cases that underwent treatment, matched to their respective biopsies from the discovery cohort, using IMC. Clinicopathological data for both cohorts are summarized in Table 1.

**Table 1:** Clinicopathological characteristics of the studied cohorts of UPS and MFS

Variable	Discovery cohort		Validation cohort	
	MFS	UPS	MFS	UPS
Total patients, *n*	17	16	22	23
Gender, *n* (%)				
Female	6 (35.3)	8 (50)	9 (40.9)	10 (43.5)
Male	12 (70.6)	8 (50)	13 (59.1)	13 (56.5)
Age (years), *median* (range)	65 (24–77)	74 (40–80)	70 (24–81)	67 (40–87)
Follow-up (months), *median* (range)	42 (6–150)	50 (12–92)	24 (4–63)	41 (15–104)
Grade, *n* (%)				
Low (1)	5 (29.4)	–	–	–
High (2, 3)	12 (70.6)	16 (100)	22 (100)	23 (100)
Anatomical location, *n* (%)				
Head & Neck	1 (5.9)	–	–	1 (4.3)
Upper extremities	3 (17.6)	1 (6.3)	3 (13.6)	4 (17.3)
Trunk	4 (23.5)	1 (6.3)	3 (13.6)	–
Lower extremities	9 (52.9)	14 (87.5)	16 (72.7)	18 (78.3)
Max tumor size (cm), *median* (IQR)^a^	7.2 (7.9)	8.9 (3.88)	10 (7.45)	8.9 (7.45)
Treatment, *n* (%)^b^				
Adjuvant radiotherapy	2 (11.8)	–	–	–
Neoadjuvant radiotherapy^c^	9 (52.9)	16 (100)	22 (100)	23 (100)
Surgery alone^d^	6 (35.3)	–	–	–
Histological response to neoadjuvant RT (%), *median* (IQR)				
Vital tumor	60 (12.5)	15 (34.3)	49.5 (45)	10 (30.5)
Hyalinization/fibrosis	20 (17.5)	40 (43.3)	15 (15)	55 (40)
Necrosis	12.5 (21.3)	20 (37.5)	25 (54.6)	20 (25)
Pathological response to neoadjuvant RT, *n* (%)^e^				
Good	–	6 (37.5)	5 (22.7)	9 (39.1)
Poor	8 (100)	10 (62.5)	17 (77.3)	14 (60.9)
Tumor depth, *n* (%)				
Deep	9 (52.9)	16 (100)	14 (63.6)	23 (100)
Superficial	8 (47.1)	–	8 (36.64)	–
Patients with recurrent disease, *n* (%)				
Local recurrence	3 (17.6)	2 (12.5)	2 (9.1)	1 (4.3)
Metastasis	3 (17.6)	8 (50)	10 (45.5)	13 (56.5)

Tumor grades were assessed according to the Fédération Nationale des Centres de Lutte Contre le Cancer (FNCLCC) grading system. IQR = interquartile range, RT = radiotherapy

a: size was missing for one UPS from the head and neck area

b: treatment for primary tumors

c: one high-grade MFS patient received neoadjuvant RT + pazopanib

d: one high-grade MFS patient had an amputation

e: pathologic response was defined as < 5% vital tumor after neoadjuvant radiotherapy

To investigate associations between clinicopathological characteristics and prognosis, we focused on a subset of 54 patients with high-grade tumors who received standard-of-care treatment, including neoadjuvant radiotherapy. In UPS, no clinical parameters, including pathologic response after neoadjuvant treatment (< 5% vital tumor), were found to associate with metastasis-free or disease-specific survival (Supplementary Table 3). In contrast, tumor size and tumor depth were identified as prognostic factors for both metastasis-free and disease-specific survival in MFS (Supplementary Table 4). Interestingly, a good pathological response to radiotherapy in MFS was negatively associated with metastasis-free and disease-specific survival.

Pathological responses to radiotherapy differed between the two sarcoma types. While UPS primarily exhibited hyalinization and fibrosis, MFS predominantly displayed necrosis (Supplementary Fig. 1a). Furthermore, histological responses in MFS were observed exclusively in large, deep-seated tumors, suggesting an association between tumor depth, tumor size, and necrosis (Supplementary Fig. 1b). Multivariate analysis did not identify any of these clinical parameters as independent prognostic factors in MFS (Supplementary Table 4).

### Heterogeneity in immune-enriched microenvironments of UPS and MFS

To evaluate the “natural” immune microenvironment of UPS and MFS, we analyzed treatment-naïve samples, including pre-treatment biopsies or surgical resections, from patients who had not received neoadjuvant radiotherapy. RNAseq was performed on 23 samples from 13 UPS and 10 MFS patients, while IMC analysis was conducted on 29 samples from 14 UPS and 15 MFS patients. Of these, 20 samples (from 11 UPS and 9 MFS patients) were included in both analyses.

To confirm that our cohort was representative of previously described immune profiles in UPS and MFS, transcriptional profiles were compared to publicly available datasets comprising 206 primary STS from TCGA [6, 22]. Immune-related transcriptional signatures were evaluated using SIC classification system [17] (Supplementary Fig. 2a) and the ICR signature [16] (Supplementary Fig. 2b).

**Fig. 1:**
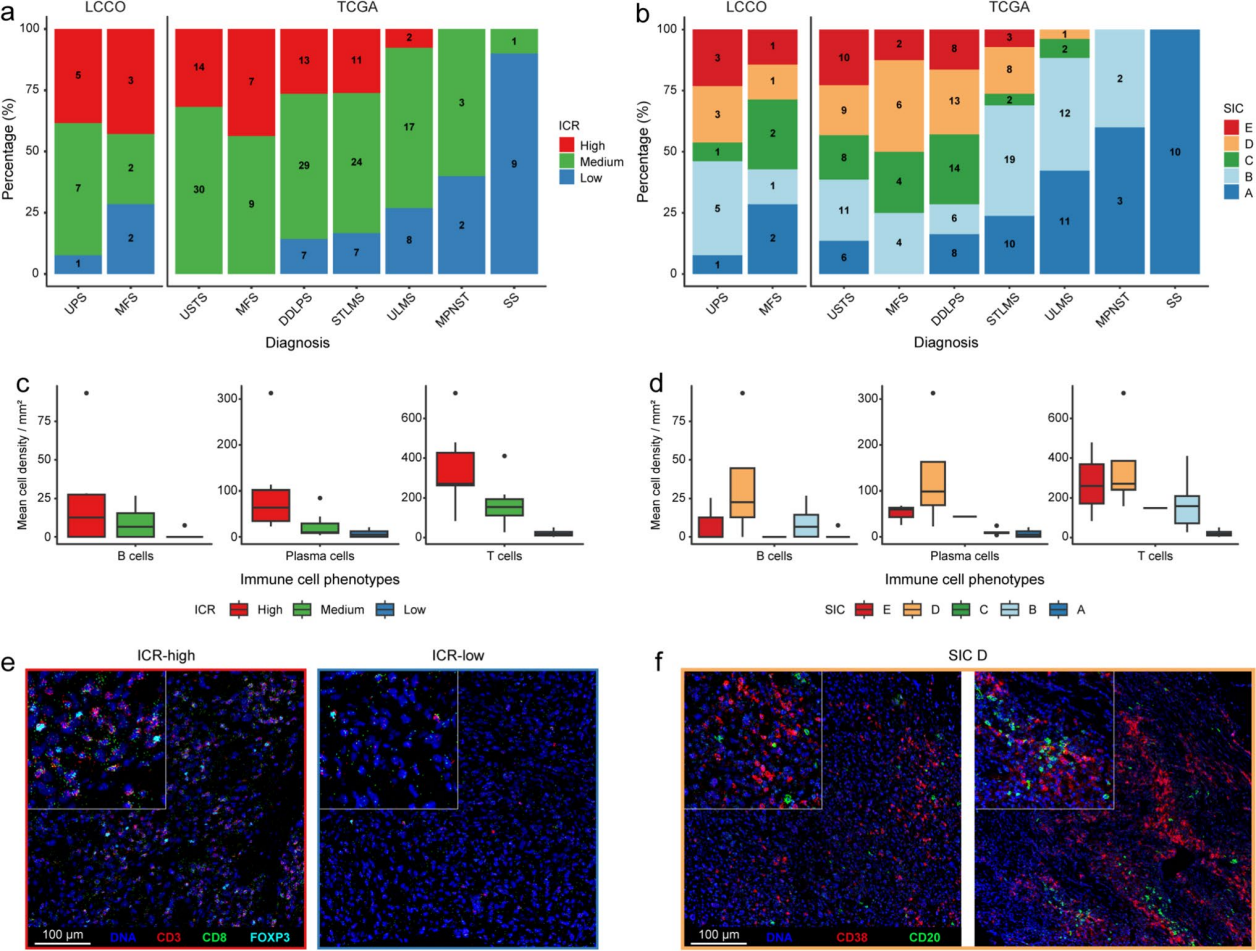
Overview of immune profiles in UPS and MFS. **a-b** Bar plots illustrating the percentage of samples across **a** ICR categories and **b)** SIC phenotypes for each STS subtype across the LCCO and TCGA datasets, excluding low-grade tumors. The number of samples in each cluster is indicated within the bars. **c-d** Boxplots presenting the IMC-based mean cell densities per mm^2^ for B cells, plasma cells and T cells per **c** ICR category and **d** SIC phenotype. **e** Representative IMC images of an ICR-high and ICR-low UPS, highlighting T cells (CD3 in red, CD8 in green, FOXP3 in cyan). **f** IMC images of UPS and MFS enriched for B cell populations (SIC D), highlighting B cells (CD20 in green) and plasma cell/plasma blasts (CD38 in red). Abbreviations: DDLPS = dedifferentiated liposarcoma; MPNST = malignant peripheral nerve sheath tumor; SS = synovial sarcoma; STLMS = soft tissue leiomyosarcoma; ULMS = uterine leiomyosarcoma

Based on the ICR signature, samples were clustered into three categories: ICR-high, ICR-medium and ICR-low [23] (Supplementary Fig. 2b). The distribution of samples across ICR and SIC categories in the LCCO cohort closely mirrored that of TCGA cohort (Fig. 1a, b), underscoring the representativeness of the LCCO cohort. UPS and high-grade MFS frequently exhibited a Th1-related immune signature compared to other STS (Fig. 1a). Up to 33% of UPS and 43% of high-grade MFS were classified as ICR-high, compared to only 0–27% in other STS (Fig. 1a). Moreover, only 2% of UPS and 9% of high-grade MFS presented as ICR-low, compared to as much as 14–90% in other STS. The SIC classification further corroborated these findings, showing an enrichment of SIC D and E classifications (immune-high) in UPS and high-grade MFS, alongside DDLPS (Fig. 1b). These classifications were much more frequent in these tumors compared to other STS (44% in UPS, 43% in high-grade MFS, 43% in DDLPS while only 0–26% in other STS).

MFS included more tumors classified as highly vascularized (SIC C) compared to UPS (26% vs 14%; Fig. 1b). However, both subtypes exhibited some heterogeneity. Up to 42% of UPS and 30% of MFS were classified as SIC A (immune-desert) or SIC B (immune-low), emphasizing that while these tumors are relatively immune-enriched, variability exists within these subtypes. In contrast, low-grade MFS (*n* = 3), which exhibit lower genomic complexity [24], displayed an immune “cold” profile overall, similar to other STS like synovial sarcoma. All three cases were classified as immune-desert (SIC A) and ICR-low (Supplementary Fig. 2a, b). These findings support the hypothesis that immunogenicity in sarcomas correlates with increasing genomic complexity [25].

Since the ICR signature reflects a Th1-related immune response and that SIC D and E phenotypes are enriched in B cell lineage signatures, we used IMC to examine the association of B cells, plasma cells, and T cells with ICR categories and SIC phenotypes (Fig. 1c, d). This analysis demonstrated a clear enrichment of these immune cell populations in ICR-high and SIC D/E phenotypes (Fig. 1e, f).

Extended immunophenotyping of UPS and MFS using IMC resulted in the identification of 32 distinct cell populations, including overlapping tumor and stromal clusters due to the absence of tumor-specific markers in these sarcomas (Supplementary Table 2). The immune cell infiltrate predominantly consisted of myeloid cells, such as macrophages and monocytes, alongside lymphoid cells, including various T cell and innate lymphoid cell populations (Supplementary Fig. 3a). Since UPS and MFS cancer cells are known to express myeloid markers like CD68 and CD163, we reviewed the imaging data to ensure that the observed myeloid cell counts were not confounded by tumor cells expressing these markers. Consistent with the immune-related transcriptional profiles, low-grade MFS exhibited the lowest immune cell densities (Supplementary Fig. 3a, b). Among high-grade tumors, UPS demonstrated higher immune cell densities compared to MFS (Supplementary Fig. 3a), which may reflect the lower overall cellularity in MFS due to their myxoid extracellular matrix.

**Fig. 2:**
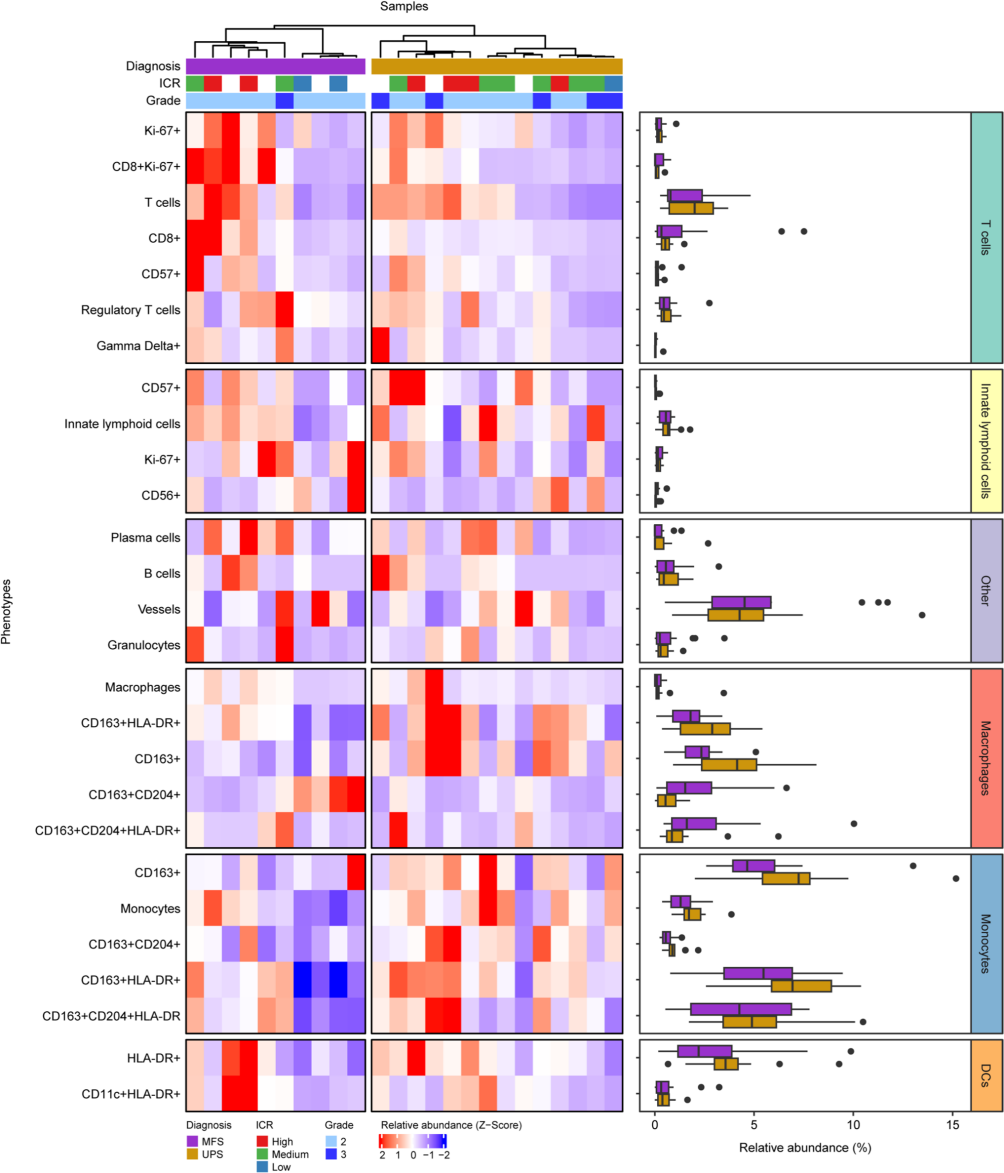
IMC-based immunophenotypic analysis of UPS and high-grade MFS. The heatmap displays the relative abundance z-scores of all identified immune cell populations within the tumor microenvironments. Samples are annotated with diagnosis, the ICR classification, and tumor grade. Missing ICR data due to unavailable RNAseq results are indicated in white. Accompanying boxplots display the relative abundance of each phenotype by tumor type. Abbreviations: DCs = dendritic cells

To correct for this difference in extracellular matrix, we calculated the relative immune cell abundance for all treatment-naïve samples of high-grade tumors (Fig. 2). Unsupervised clustering revealed a relative enrichment of T cells in a subset of both MFS and UPS (Fig. 2; Supplementary Fig. 3c), while myeloid cells, including monocytes and macrophages, were more prevalent in UPS compared to MFS (Fig. 2).

### T cell and CD68^+^CD163^+^ macrophage infiltration is associated with an improved prognosis in UPS but not in MFS

To validate the IMC findings and investigate the clinical impact of T and myeloid cell populations, we composed a cohort of 45 patients (23 UPS and 22 high-grade MFS) that underwent the current standard-of-care treatment of neoadjuvant radiotherapy followed by surgery. T cell infiltration was assessed in pre-treatment biopsies using a multispectral IF panel, including anti-CD3, anti-CD8 and anti-PD-1 antibodies, while macrophages were evaluated with anti-CD68 and anti-CD163 antibodies by two-color IHC. Based on the IMC findings, CD163 was included to identify most macrophages and the dual staining with CD68 supported the distinction between macrophages and tumor cells.

**Fig. 3:**
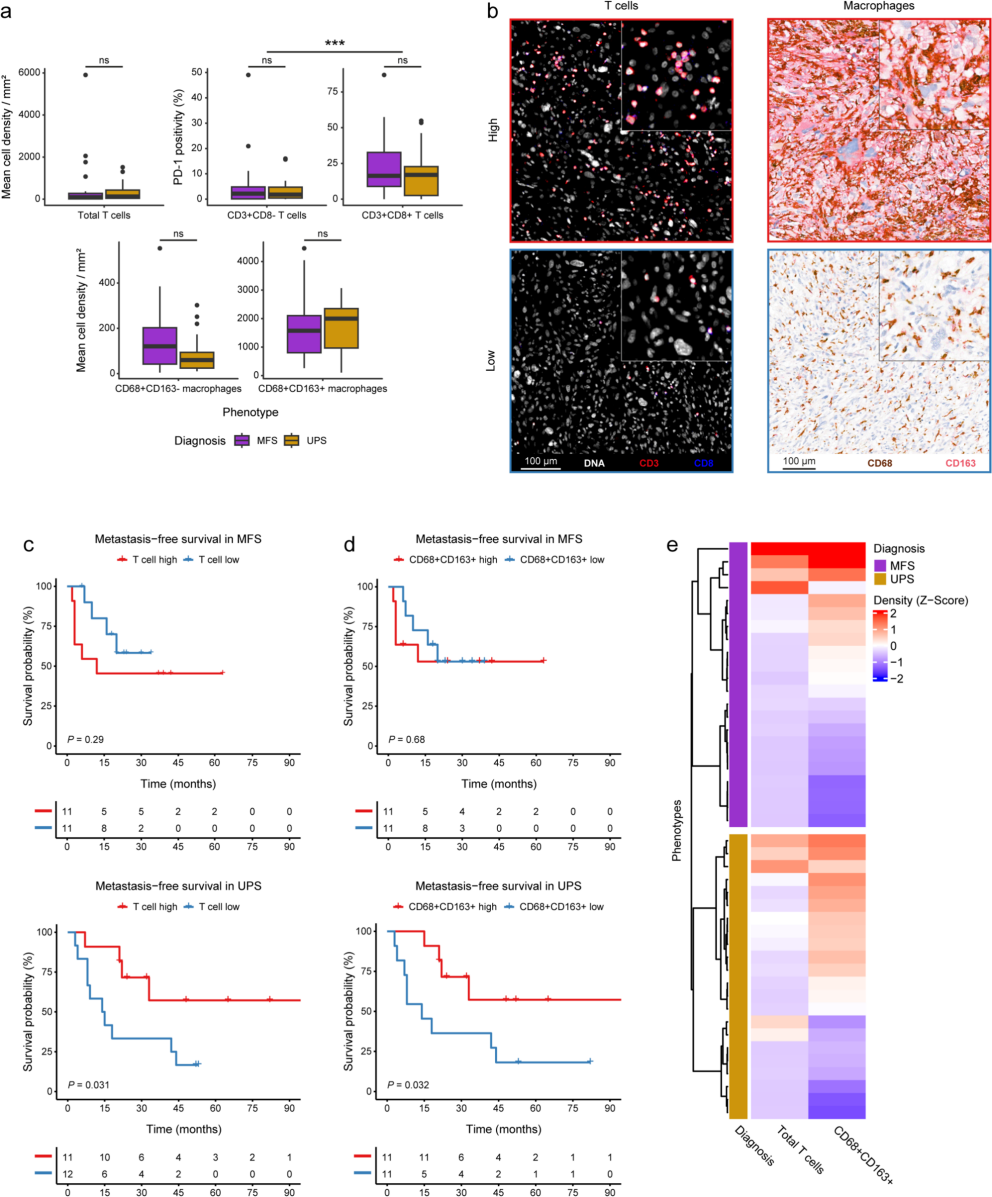
T cells and CD68^+^CD163^+^ macrophages are prognostic in UPS, but not in MFS. **a** Boxplots presenting the mean cell densities per mm^2^ of T cells and macrophages, as well as the percentage of PD-1^+^ T cells (CD8^−^ and CD8^+^) in the validation cohort. The significance level was evaluated with a Student’s t test and is indicated per phenotype. ns = not significant, *** = *P* < 0.001. **b** Representative IF images of a T cell high and a T cell low UPS (CD3 in red, CD8 in blue) and double IHC images of a CD68^+^CD163^+^ high and a CD68^+^CD163^+^ low MFS (CD68 in brown, CD163 in magenta). **c**, **d** Survival curves for metastasis-free survival in both MFS and UPS, **c** grouped based on the median T cell infiltration and **d** grouped based on the median CD68^+^CD163^+^ macrophage infiltration. The association between macrophages and metastasis-free survival could not be assessed for one UPS patient due to insufficient biopsy material for macrophage detection. Significance is presented per Kaplan–Meier curve by the log-rank *P* value. **e** Heatmap showing the z-score of the mean cell densities per mm^2^ of T cells and CD68^+^CD163^+^ macrophages in UPS and MFS, as identified by IF and IHC, respectively. The samples are annotated according to their diagnosis

Consistent with the IMC results, T cell infiltration showed high variability in both UPS and MFS (Fig. 3a). A strong correlation was observed between T cell infiltration levels determined by IMC and those detected using multispectral IF (Supplementary Fig. 4a). A significantly larger proportion of CD8^+^ T cells exhibited PD-1 positivity (min: 0%, median: ~ 17%, max: ~ 87%) compared to CD8^−^ T cells (supposedly CD4^+^ T cells; min: 0%, median: ~ 2%, max: ~ 49%; *P* = 4.1e-6), with similar levels in UPS and MFS (Fig. 3a). For survival analysis, the cohort was grouped into T cell high and T cell low based on the median total T cell density per mm^2^ within each subtype (Fig. 3a, b). Higher T cell infiltration was associated with improved metastasis-free survival in UPS but not in MFS (Fig. 3c). The same association was observed for disease-specific survival, but was not found statistically significant due to the low number of events (Supplementary Fig. 4b).

For the macrophages, both UPS and MFS exhibited comparable densities of CD68^+^CD163^+^ macrophages, while MFS displayed higher levels of CD68^+^CD163^−^ macrophages, though this difference was not statistically significant (Fig. 3a). As with T cells, samples were categorized as macrophage high or low based on the median macrophage density per mm^2^, which was assessed separately for CD68^+^CD163^−^ and CD68^+^CD163^+^ subsets. CD68^+^CD163^+^ macrophages associated with better metastasis-free survival in UPS but not in MFS (Fig. 3d). Again, the same association was observed for disease-specific survival and not found statistically significant due to the low number of events (Supplementary Fig. 4b). Interestingly, high T cell infiltration was linked to high CD68^+^CD163^+^ macrophage infiltration in both subtypes (Fig. 3e), suggesting a coordinated role of these immune cells within the tumor microenvironment. This was further strengthened by the observation that patients with high infiltration of both T cell and CD68^+^CD163^+^ macrophages had the longest metastasis-free survival, whereas patients with low infiltration of both immune phenotypes had the shortest metastasis-free survival in UPS (Supplementary Fig. 4c). CD68^+^CD163^−^ macrophages were not associated with survival in either subtype (Supplementary Fig. 4d). Additionally, the ratio of T cells to CD68^+^CD163^+^ macrophages was assessed for both UPS and MFS and analyzed in relation to metastasis-free survival (Supplementary Fig. 4e, f). Although not statistically significant, an opposite association between the T cell-to-macrophage ratio and metastasis-free survival was observed for UPS and MFS, further underscoring the diverging roles of the immune microenvironment in these sarcoma subtypes.

### Neoadjuvant radiation affects UPS and MFS differently

**Fig. 4:**
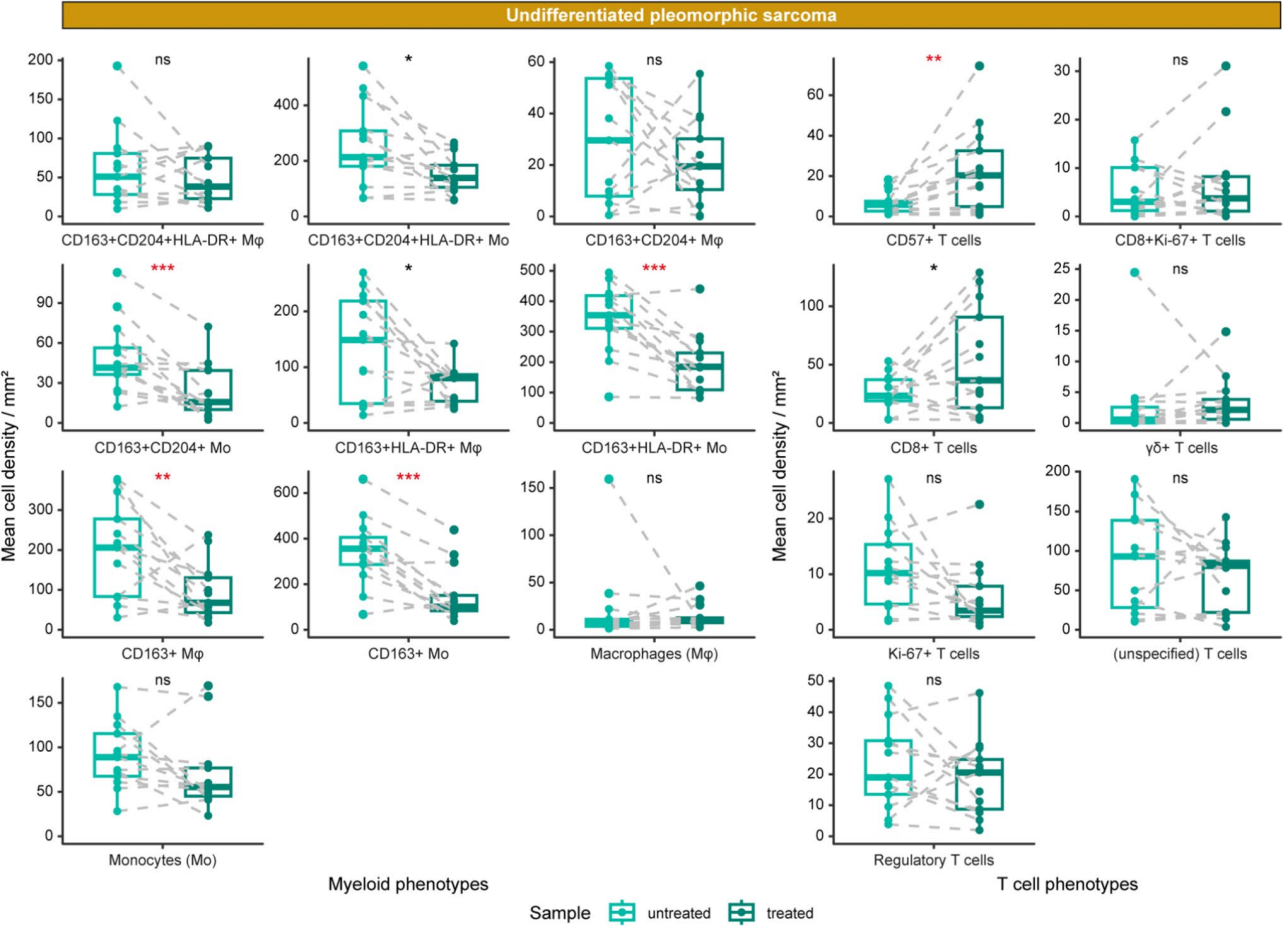
The effect of radiotherapy on UPS. IMC-based comparison of pre- vs post-treatment UPS, presented in paired boxplots. Immune cell phenotypes are grouped into myeloid and T cell phenotypes. Myeloid phenotypes include monocytes (Mο; CD14^+^CD68^−^) and macrophages (Mφ; CD68.^+^). The significance level was evaluated with a Student’s t test followed by a Benjamini–Hochberg false discovery rate (FDR) correction. The significance is indicated per phenotype and FDR-significant phenotypes are indicated in red. ns = not significant, * = *P* < 0.05, ** = *P* < 0.01, *** = *P* < 0.001

Since T cells and macrophages were assessed in pre-treatment samples for their prognostic value—and that neoadjuvant radiotherapy is part of the standard-of-care for both UPS and MFS—we investigated whether these immune cell populations respond differently to radiotherapy in the two subtypes. IMC data was used to compare matched pre- and post-treatment samples of 21 patients (13 UPS and 8 MFS). In post-treatment samples, only vital tumor areas were assessed, including both central and peripheral regions to account for intratumoral heterogeneity. In UPS (*n* = 13), various myeloid cell populations decreased following radiotherapy (Fig. 4), including both monocytes (CD14^+^CD68^−^) and macrophages (CD68^+^). Among T cells, CD57^+^ T cells and CD8^+^ T cells increased after radiotherapy (*P* < 0.01 and *P* < 0.05, respectively). Additional phenotypes affected by the radiotherapy in UPS included tumor/stromal cell populations and vessels, which also decreased (Supplementary Fig. 5a). These alterations were all independent from pathological response. In contrast, no significant alterations in the immune microenvironment were observed in MFS following neoadjuvant radiotherapy (*n* = 8; Supplementary Fig. 5a, b). These findings suggest that radiotherapy may exert an immunogenic effect specifically in UPS.

## Discussion

Although both UPS and MFS demonstrate more hallmarks of anti-tumor immunity than other STS, this study reveals considerable differences between the two subtypes. While both subtypes include T cell-enriched tumors, UPS demonstrated relatively higher myeloid cell infiltration compared to MFS. In UPS, T cells and CD68^+^CD163^+^ macrophages were positively associated with improved metastasis-free survival. Interestingly, radiotherapy altered the UPS microenvironment, reducing myeloid cell populations and increasing cytotoxic T cell phenotypes. In contrast, in MFS, neither T cells nor macrophages were associated with survival, and the immune microenvironment remained largely unchanged following radiotherapy. These findings highlight fundamental biological differences between the two subtypes, with UPS being more responsive to immune modulation by radiotherapy.

There is a growing interest in the immune microenvironment of STS, particularly UPS, due to its responsiveness to T cell checkpoint blockade [26–28]. While pathological response to neoadjuvant radiotherapy was not prognostic in UPS, consistent with the findings from Danieli et al*.* [29], a pre-existing immune response appeared to reduce the metastatic potential of tumors. Furthermore, we show that radiotherapy may promote a more favorable context for anti-tumor immunity in UPS, potentially enhancing T cell-mediated responses and modulating myeloid cell interactions. These observations appear to align with findings by Keung et al*.* [30], who observed a trend for increased T cell infiltration following radiotherapy in UPS. Interestingly, our findings contrast with the ones by Goff et al*.,* who reported an increase in myeloid cells after radiotherapy in UPS [31]. This discrepancy may arise from differences in data presentation; Goff et al. reported cell percentages relative to total cells, whereas we analyzed cell densities per tissue area to enable accurate pre- and post-treatment comparisons. Despite these differences, our results underscore the potential of radiotherapy to enhance anti-tumor immunity in UPS.

The association between CD68^+^CD163^+^ macrophages and metastasis-free survival highlights the complexity of macrophage biology, given that CD163^+^ macrophages have traditionally been associated with worse outcomes in many solid tumors [32], including various sarcoma subtypes [33–35]. However, several studies on sarcomas, including UPS, have reported positive associations with clinical outcomes [36–39], emphasizing the context-dependent nature of macrophage function. Moreover, we observed a marked decrease in their abundance following neoadjuvant radiotherapy, further complicating the interpretation of their role. Given the well-established functional heterogeneity of tumor-associated macrophages [40], further research will be needed to define the specific roles and functional states of these populations in UPS. Although the current study highlights the prognostic effect of immunity in the context of neoadjuvant radiotherapy, it is important to note that this effect may be independent of radiotherapy altogether, as studies by Toulmonde et al*.* and Guegan et al*.* found CD8^+^ T cells to be prognostic in both untreated and chemotherapy-treated UPS, respectively [36, 41].

Similar to UPS, MFS has shown responsiveness to T cell checkpoint blockade [42], suggesting that anti-tumor immune responses can also be mounted in these tumors. However, our findings do not support a strong modulatory effect of radiotherapy in the immune microenvironment of MFS, and pre-existing immunity appears to lack prognostic significance. This aligns with the findings of Yamashita et al*.* [24], who also demonstrated that T cell infiltration is not prognostic in MFS. The divergence between the roles of the immune microenvironment in UPS and MFS is striking. Despite similar overall immune cell densities, functional differences or distinct tumor–immune interactions likely drive these outcomes. For instance, Dancsok et al*.* identified SIRPα^+^ macrophages as negative prognostic markers specific to MFS and not in UPS [33]. Furthermore, the lack of significant immune alterations in MFS post-radiotherapy underscores these differences. Future research could provide deeper insights into the functional heterogeneity and dynamics of the immune microenvironment of UPS and MFS.

Neoadjuvant radiotherapy together with surgical resection effectively controlled disease in both UPS and MFS, with only 4 out of 54 high-grade tumors experiencing local recurrence. However, metastasis remains a major challenge, occurring in approximately 50% of patients across both subtypes. This highlights the need for additional systemic therapies to target circulating or metastasizing tumor cells. Promisingly, Roland et al*.* [43] and Mowery et al*.* [44] demonstrated survival benefits with the combination of neoadjuvant checkpoint blockade and radiotherapy in UPS. For cases lacking a pre-existing immune response, converting the microenvironment from “cold” to “hot” might be necessary. Guegan et al*.* observed that immune “cold” UPS responded well to neoadjuvant chemotherapy [36], which subsequently enhanced immune cell infiltration.

Our study has several limitations, including a relatively small sample size and a single-institution design, with all patients receiving neoadjuvant radiotherapy. Most samples were diagnostic biopsies, which limited tissue availability. Furthermore, treatment approaches have only recently become more standardized for UPS and MFS. As a result, we were unable to establish independent discovery and validation cohorts, Nevertheless, this study provides important insights into the immune landscape of these rare sarcomas and highlights key differences between these UPS and MFS, offering a foundation for future research.

In conclusion, this study underscores key differences in the immune microenvironments of UPS and MFS. While both subtypes exhibited high immune cell infiltration, only UPS showed improved metastasis-free survival associated with T cells and CD68^+^CD163^+^ macrophages. Radiotherapy appeared to reshape the immune response in UPS, increasing cytotoxic T cell infiltration and reducing myeloid cell populations, yet MFS remained largely resistant to such modulation. These results suggest that subtype-specific approaches may be required to improve outcomes from cancer immunotherapy in STS.

## Supplementary Information

Below is the link to the electronic supplementary material.

Supplementary file1 (PDF 7742 KB)

## Data Availability

The RNAseq data generated in this study are publicly available in Gene Expression Omnibus GEO at GSE285944. The IMC data is available in BioStudies at S-BIAD1555. The QuPath script and the R code used to process the data are published on the LUMC Bone and Soft Tissue Pathology Group GitLab (https://git.lumc.nl/bstp/papers/immunogenic-features-of-ups-and-mfs). All other data are available from the corresponding author upon reasonable request.

## References

[1] WHO Classification of Tumours Editorial Board (2020) Soft Tissue and Bone Tumours. WHO Classification of Tumours. 5 ed. Lyon, France: IARC Publications

[2] Gronchi A, Miah AB, Dei Tos AP, Abecassis N, Bajpai J, Bauer S et al (2021) Soft tissue and visceral sarcomas: ESMO-EURACAN-GENTURIS clinical practice guidelines for diagnosis, treatment and follow-up. Ann Oncol 32(11):1348–1365 34303806 10.1016/j.annonc.2021.07.006

[3] Yoshimoto M, Yamada Y, Ishihara S, Kohashi K, Toda Y, Ito Y et al (2020) Comparative study of myxofibrosarcoma with undifferentiated pleomorphic sarcoma: histopathologic and clinicopathologic review. Am J Surg Pathol 44(1):87–97 31651522 10.1097/PAS.0000000000001389

[4] van der Horst CAJ, Bongers SLM, Versleijen-Jonkers YMH, Ho VKY, Braam PM, Flucke UE et al (2022) Overall survival of patients with myxofibrosarcomas: an epidemiological study. Cancers (Basel). 10.3390/cancers14051102 10.3390/cancers14051102 PMC8909833 35267410

[5] Gonzalez MR, Clunk MJ, Bedi ADS, Werenski JO, Lang JH, Karczewski D et al (2023) Prognostic and predictive factors in undifferentiated pleomorphic sarcoma: a long-term study from a large tertiary care urban center. J Surg Oncol 128(2):322–331 37042427 10.1002/jso.27279

[6] Lazar AJ, McLellan MD, Bailey MH, Miller CA, Appelbaum EL, Cordes MG, Lichtenberg TM (2017) Comprehensive and integrated genomic characterization of adult soft tissue sarcomas. Cell 171(4):950–965 29100075 10.1016/j.cell.2017.10.014 PMC5693358

[7] Mitra S, Farswan A, Piccinelli P, Sydow S, Hesla A, Tsagkozis P et al (2024) Transcriptomic profiles of myxofibrosarcoma and undifferentiated pleomorphic sarcoma correlate with clinical and genomic features. J Pathol 264(3):293–304 39258383 10.1002/path.6347

[8] Lyskjaer I, De Noon S, Tirabosco R, Rocha AM, Lindsay D, Amary F et al (2021) DNA methylation-based profiling of bone and soft tissue tumours: a validation study of the ‘DKFZ sarcoma classifier.’ J Pathol Clin Res 7(4):350–360 33949149 10.1002/cjp2.215 PMC8185366

[9] Koelsche C, Schrimpf D, Stichel D, Sill M, Sahm F, Reuss DE et al (2021) Sarcoma classification by DNA methylation profiling. Nat Commun 12(1):498 33479225 10.1038/s41467-020-20603-4 PMC7819999

[10] Allignet B, Meurgey A, Bouhamama A, Karanian M, Meeus P, Vaz G et al (2021) Impact of histological subtype on radiological and pathological response after neoadjuvant radiotherapy in soft tissue sarcoma. Eur J Surg Oncol 47(12):2995–3003 34281731 10.1016/j.ejso.2021.07.008

[11] Messiou C, Bonvalot S, Gronchi A, Vanel D, Meyer M, Robinson P et al (2016) Evaluation of response after pre-operative radiotherapy in soft tissue sarcomas; the european organisation for research and treatment of cancer-soft tissue and bone sarcoma group (EORTC-STBSG) and imaging group recommendations for radiological examination and reporting with an emphasis on magnetic resonance imaging. Eur J Cancer 56:37–44 26802529 10.1016/j.ejca.2015.12.008

[12] Schaefer IM, Hornick JL, Barysauskas CM, Raut CP, Patel SA, Royce TJ et al (2017) Histologic appearance after preoperative radiation therapy for soft tissue sarcoma: assessment of the European organization for research and treatment of cancer-soft tissue and bone sarcoma group response score. Int J Radiat Oncol Biol Phys 98(2):375–383 28463157 10.1016/j.ijrobp.2017.02.087

[13] van Oost S, Meijer DM, Ijsselsteijn ME, Roelands JP, van den Akker B, van der Breggen R et al (2024) Multimodal profiling of chordoma immunity reveals distinct immune contextures. J Immunother Cancer 12(1):e008138 38272563 10.1136/jitc-2023-008138 PMC10824073

[14] Anders S, Pyl PT, Huber W (2015) HTSeq–a Python framework to work with high-throughput sequencing data. Bioinformatics 31(2):166–169 25260700 10.1093/bioinformatics/btu638 PMC4287950

[15] Bertucci F, Finetti P, Simeone I, Hendrickx W, Wang E, Marincola FM et al (2018) The immunologic constant of rejection classification refines the prognostic value of conventional prognostic signatures in breast cancer. Br J Cancer 119(11):1383–1391 30353048 10.1038/s41416-018-0309-1 PMC6265245

[16] Bertucci F, Niziers V, de Nonneville A, Finetti P, Mescam L, Mir O et al (2022) Immunologic constant of rejection signature is prognostic in soft-tissue sarcoma and refines the CINSARC signature. J Immunother Cancer. 10.1136/jitc-2021-003687 10.1136/jitc-2021-003687 PMC8753443 35017155

[17] Petitprez F, de Reynies A, Keung EZ, Chen TW, Sun CM, Calderaro J et al (2020) B cells are associated with survival and immunotherapy response in sarcoma. Nature 577(7791):556–560 31942077 10.1038/s41586-019-1906-8

[18] Ijsselsteijn ME, van der Breggen R, Sarasqueta AF, Koning F, de Miranda NFCC (2019) A 40-marker panel for high dimensional characterization of cancer immune microenvironments by imaging mass cytometry. Front Immunol 10:484562 10.3389/fimmu.2019.02534 PMC6830340 31736961

[19] Ijsselsteijn ME, Somarakis A, Lelieveldt BPF, Hollt T, de Miranda N (2021) Semi-automated background removal limits data loss and normalizes imaging mass cytometry data. Cytometry A 99(12):1187–1197 34196108 10.1002/cyto.a.24480 PMC9542015

[20] Somarakis A, Van Unen V, Koning F, Lelieveldt B, Hollt T (2021) ImaCytE: visual exploration of cellular micro-environments for imaging mass cytometry data. IEEE Trans Vis Comput Graph 27(1):98–110 31369380 10.1109/TVCG.2019.2931299

[21] Ijsselsteijn ME, Brouwer TP, Abdulrahman Z, Reidy E, Ramalheiro A, Heeren AM et al (2019) Cancer immunophenotyping by seven-colour multispectral imaging without tyramide signal amplification. J Pathol Clin Res 5(1):3–11 30191683 10.1002/cjp2.113 PMC6317065

[22] van IJzendoorn DGP, Szuhai K, Briaire-de Bruijn IH, Kostine M, Kuijjer ML, Bovee JVMG (2019) Machine learning analysis of gene expression data reveals novel diagnostic and prognostic biomarkers and identifies therapeutic targets for soft tissue sarcomas. PLoS Comput Biol 15(2):e1006826 30785874 10.1371/journal.pcbi.1006826 PMC6398862

[23] Roelands J, Hendrickx W, Zoppoli G, Mall R, Saad M, Halliwill K et al (2020) Oncogenic states dictate the prognostic and predictive connotations of intratumoral immune response. J Immunother Cancer. 10.1136/jitc-2020-000617 10.1136/jitc-2020-000617 PMC7223637 32376723

[24] Yamashita A, Suehara Y, Hayashi T, Takagi T, Kubota D, Sasa K et al (2022) Molecular and clinicopathological analysis revealed an immuno-checkpoint inhibitor as a potential therapeutic target in a subset of high-grade myxofibrosarcoma. Virchows Arch 481(4):1–17 10.1007/s00428-022-03358-9 35705750

[25] van Oost S, Meijer DM, Kuijjer ML, Bovee J, de Miranda N (2021) Linking immunity with genomics in sarcomas: Is genomic complexity an immunogenic trigger? Biomedicines 9(8):1048 34440251 10.3390/biomedicines9081048 PMC8391750

[26] Tawbi HA, Burgess M, Bolejack V, Van Tine BA, Schuetze SM, Hu J et al (2017) Pembrolizumab in advanced soft-tissue sarcoma and bone sarcoma (SARC028): a multicentre, two-cohort, single-arm, open-label, phase 2 trial. Lancet Oncol 18(11):1493–1501 28988646 10.1016/S1470-2045(17)30624-1 PMC7939029

[27] Italiano A, Bessede A, Pulido M, Bompas E, Piperno-Neumann S, Chevreau C et al (2022) Pembrolizumab in soft-tissue sarcomas with tertiary lymphoid structures: a phase 2 PEMBROSARC trial cohort. Nat Med 28(6):1199–1206 35618839 10.1038/s41591-022-01821-3

[28] Keung EZ, Burgess M, Salazar R, Parra ER, Rodrigues-Canales J, Bolejack V et al (2020) Correlative analyses of the SARC028 trial reveal an association between sarcoma-associated immune infiltrate and response to pembrolizumab. Clin Cancer Res 26(6):1258–1266 31900276 10.1158/1078-0432.CCR-19-1824 PMC7731262

[29] Danieli M, Barretta F, Radaelli S, Fiore M, Sangalli C, Barisella M et al (2023) Pathological and radiological response following neoadjuvant treatments in primary localized resectable myxofibrosarcoma and undifferentiated pleomorphic sarcoma of the extremities and trunk wall. Cancer 129(21):3417–3429 37452607 10.1002/cncr.34945

[30] Keung EZ, Tsai JW, Ali AM, Cormier JN, Bishop AJ, Guadagnolo BA et al (2018) Analysis of the immune infiltrate in undifferentiated pleomorphic sarcoma of the extremity and trunk in response to radiotherapy: rationale for combination neoadjuvant immune checkpoint inhibition and radiotherapy. Oncoimmunology 7(2):e1385689 29308306 10.1080/2162402X.2017.1385689 PMC5749668

[31] Goff PH, Riolobos L, LaFleur BJ, Spraker MB, Seo YD, Smythe KS et al (2022) Neoadjuvant therapy induces a potent immune response to sarcoma, dominated by myeloid and B cells. Clin Cancer Res 28(8):1701–1711 35115306 10.1158/1078-0432.CCR-21-4239 PMC9953754

[32] Mathiesen H, Juul-Madsen K, Tramm T, Vorup-Jensen T, Moller HJ, Etzerodt A et al (2025) Prognostic value of CD163(+) macrophages in solid tumor malignancies: a scoping review. Immunol Lett 272:106970 39778658 10.1016/j.imlet.2025.106970

[33] Dancsok AR, Gao D, Lee AF, Steigen SE, Blay JY, Thomas DM et al (2020) Tumor-associated macrophages and macrophage-related immune checkpoint expression in sarcomas. Oncoimmunology 9(1):1747340 32313727 10.1080/2162402X.2020.1747340 PMC7153829

[34] Nystrom H, Jonsson M, Nilbert M, Carneiro A (2023) Immune-cell infiltration in high-grade soft tissue sarcomas; prognostic implications of tumor-associated macrophages and B-cells. Acta Oncol 62(1):33–39 36786033 10.1080/0284186X.2023.2172688

[35] Umakoshi M, Nakamura A, Tsuchie H, Li Z, Kudo-Asabe Y, Miyabe K et al (2023) Macrophage numbers in the marginal area of sarcomas predict clinical prognosis. Sci Rep 13(1):1290 36690825 10.1038/s41598-023-28024-1 PMC9870999

[36] Guegan JP, El Ghazzi N, Vibert J, Rey C, Vanhersecke L, Coindre JM et al (2024) Predictive value of tumor microenvironment on pathologic response to neoadjuvant chemotherapy in patients with undifferentiated pleomorphic sarcomas. J Hematol Oncol 17(1):100 39444039 10.1186/s13045-024-01614-w PMC11515614

[37] Lazcano R, Barreto CM, Salazar R, Carapeto F, Traweek RS, Leung CH et al (2022) The immune landscape of undifferentiated pleomorphic sarcoma. Front Oncol 12:1008484 36313661 10.3389/fonc.2022.1008484 PMC9597628

[38] Buddingh EP, Kuijjer ML, Duim RA, Burger H, Agelopoulos K, Myklebost O et al (2011) Tumor-infiltrating macrophages are associated with metastasis suppression in high-grade osteosarcoma: a rationale for treatment with macrophage activating agents. Clin Cancer Res 17(8):2110–2119 21372215 10.1158/1078-0432.CCR-10-2047

[39] Gomez-Brouchet A, Illac C, Gilhodes J, Bouvier C, Aubert S, Guinebretiere JM et al (2017) CD163-positive tumor-associated macrophages and CD8-positive cytotoxic lymphocytes are powerful diagnostic markers for the therapeutic stratification of osteosarcoma patients: an immunohistochemical analysis of the biopsies fromthe French OS2006 phase 3 trial. Oncoimmunology 6(9):e1331193 28932633 10.1080/2162402X.2017.1331193 PMC5599091

[40] Ma RY, Black A, Qian BZ (2022) Macrophage diversity in cancer revisited in the era of single-cell omics. Trends Immunol 43(7):546–563 35690521 10.1016/j.it.2022.04.008

[41] Toulmonde M, Lucchesi C, Verbeke S, Crombe A, Adam J, Geneste D et al (2020) High throughput profiling of undifferentiated pleomorphic sarcomas identifies two main subgroups with distinct immune profile, clinical outcome and sensitivity to targeted therapies. EBioMedicine 62:103131 33254023 10.1016/j.ebiom.2020.103131 PMC7708794

[42] D’Angelo SP, Mahoney MR, Van Tine BA, Atkins J, Milhem MM, Jahagirdar BN et al (2018) Nivolumab with or without ipilimumab treatment for metastatic sarcoma (Alliance A091401): two open-label, non-comparative, randomised, phase 2 trials. Lancet Oncol 19(3):416–426 29370992 10.1016/S1470-2045(18)30006-8 PMC6126546

[43] Roland CL, Nassif Haddad EF, Keung EZ, Wang WL, Lazar AJ, Lin H et al (2024) A randomized, non-comparative phase 2 study of neoadjuvant immune-checkpoint blockade in retroperitoneal dedifferentiated liposarcoma and extremity/truncal undifferentiated pleomorphic sarcoma. Nat Cancer. 10.1038/s43018-024-00726-z 10.1038/s43018-024-00726-z PMC12955605 38351182

[44] Mowery YM, Ballman KV, Hong AM, Schuetze SM, Wagner AJ, Monga V et al (2024) Safety and efficacy of pembrolizumab, radiation therapy, and surgery versus radiation therapy and surgery for stage III soft tissue sarcoma of the extremity (SU2C-SARC032): an open-label, randomised clinical trial. Lancet 404(10467):2053–2064 39547252 10.1016/S0140-6736(24)01812-9 PMC11842127

