# Supplementary material for: Divergent therapeutic and prognostic impacts of immunogenic features in undifferentiated pleomorphic sarcoma and myxofibrosarcoma
Source: Cancer Immunol Immunother. 2025 Jul 2;74(8):258. doi: 10.1007/s00262-025-04123-y (PMC12222583; doi:10.1007/s00262-025-04123-y)
Supplement: Supplementary file 1 — Supplementary file1 (PDF 7742 KB) [file 262_2025_4123_MOESM1_ESM.pdf]

Index of supplemental information

Supplementary Methods | QuPath workflow to analyze double IHC images

Supplementary Fig. 1 | Association between tumor depth, tumor size and necrosis in MFS

Supplementary Fig. 2 | Clustering using the SIC and ICR signatures in UPS and MFS

Supplementary Fig. 3 | Overview of immune contextures in UPS and MFS

Supplementary Fig. 4 | Association between immune infiltration and survival in UPS and MFS

Supplementary Fig. 5 | The effect of radiotherapy on UPS and MFS

Supplementary Table 1 | IMC marker panel

Supplementary Table 2 | Cell types and used lineage markers for the IMC analysis

Supplementary Table 3 | Univariate cox proportional hazard results for disease-specific and metastasis-free survival in UPS

Supplementary Table 4 | Univariate and multivariate cox proportional hazard results for disease-specific and metastasis-free survival in MFS

Supplementary Methods

QuPath workflow to analyze double IHC images

- Load images as Brightfield (other).

- Single stains for CD68-DAB and CD163-AP were performed to identify the color values (estimate stain vectors in QuPath).

- Set deconvolution stain values manually based on the single stains.

- Create ROIs in each image (~ 1x1.3 mm rectangles).

- Detect (mostly) macrophages by using the optical density sum (instead of hematoxylin), this takes the intensity of the staining in consideration to detect cells rather than the hematoxylin. As depicted below, the result is that tumor cells (with the large nuclei) are often not even “detected” since the DAB staining is much stronger in macrophages. Moreover, this allows for a more proper identification of the macrophages their shape rather than taking the nucleus of a cell and then expanding with x µm. Using this cell segmentation method, you lose some accuracy in detecting the exact number of cells (some cells are oversegmented, others are undersegmented), but it allows for a more robust separation of tumor cells and macrophages and is consistent across the cohort.

- Train an object classifier to classify cells as either CD68+, CD163+, CD68+CD163+ or negative.
    - CD68+ cells and CD68+CD163+ cells were considered macrophages, while CD163+ cells were considered tumor cells and therefore excluded.

- Measure tissue area per ROI by training a pixel classifier (ANN_MLP or RTrees).

- Save detection measurements (cells / ROI) and annotation measurements (tissue area / ROI).

- In R -> count cells per ROI, then correct for tissue area, then average per sample.

- The used QuPath & R scripts are available on our GitLab.

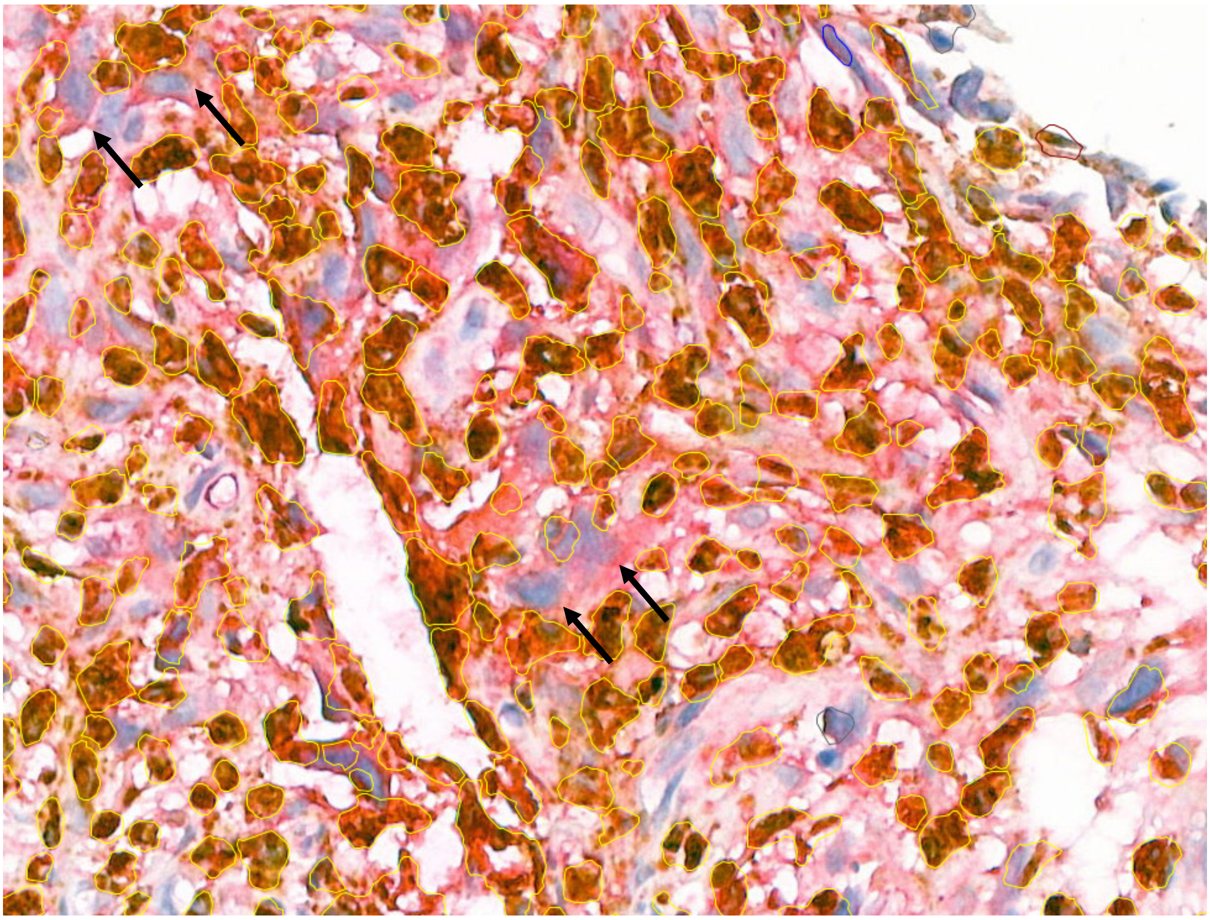

Snapshot of a double IHC image of an MFS. Macrophages were stained for CD68 (DAB-brown) and CD163 (AP-magenta). Cell detection is shown by the polygonal shapes. Yellow = $CD68^{+}CD163^{+}$, Blue = $CD163^{+}$, Brown = $CD68^{+}$. Black arrows indicate $CD68^{+}$ and/or $CD163^{+}$ tumor cells.

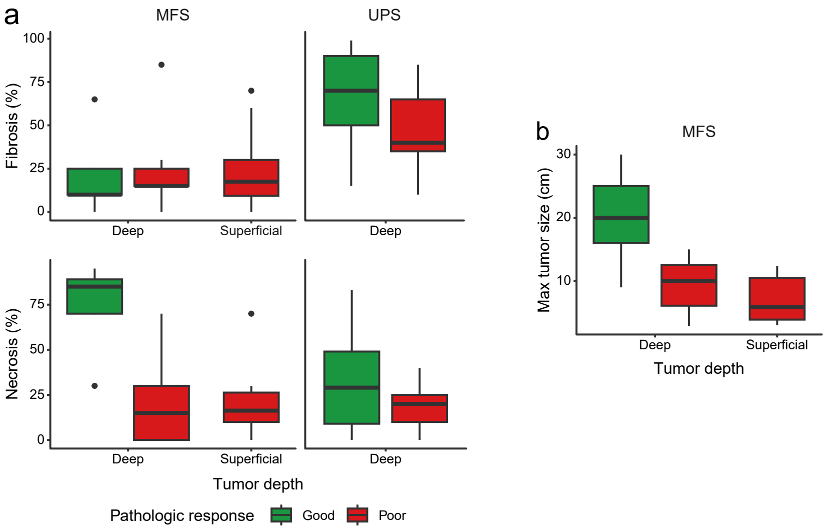
a
MFS
UPS
Fibrosis (%)
100
75
50
25
0
Deep
Superficial
Deep
Necrosis (%)
75
50
25
0
Deep
Superficial
Deep
Tumor depth
Pathologic response
Good
Poor
b
MFS
Max tumor size (cm)
30
20
10
Deep
Superficial
Tumor depth

Supplementary Fig. 1 Association between tumor depth, tumor size and necrosis in MFS. **a)** Boxplots displaying the association between pathologic response (<%5 vital tumor), fibrosis, necrosis, tumor depth and diagnosis. **b)** Boxplots presenting the association between max tumor size (cm), pathologic response and tumor depth in MFS

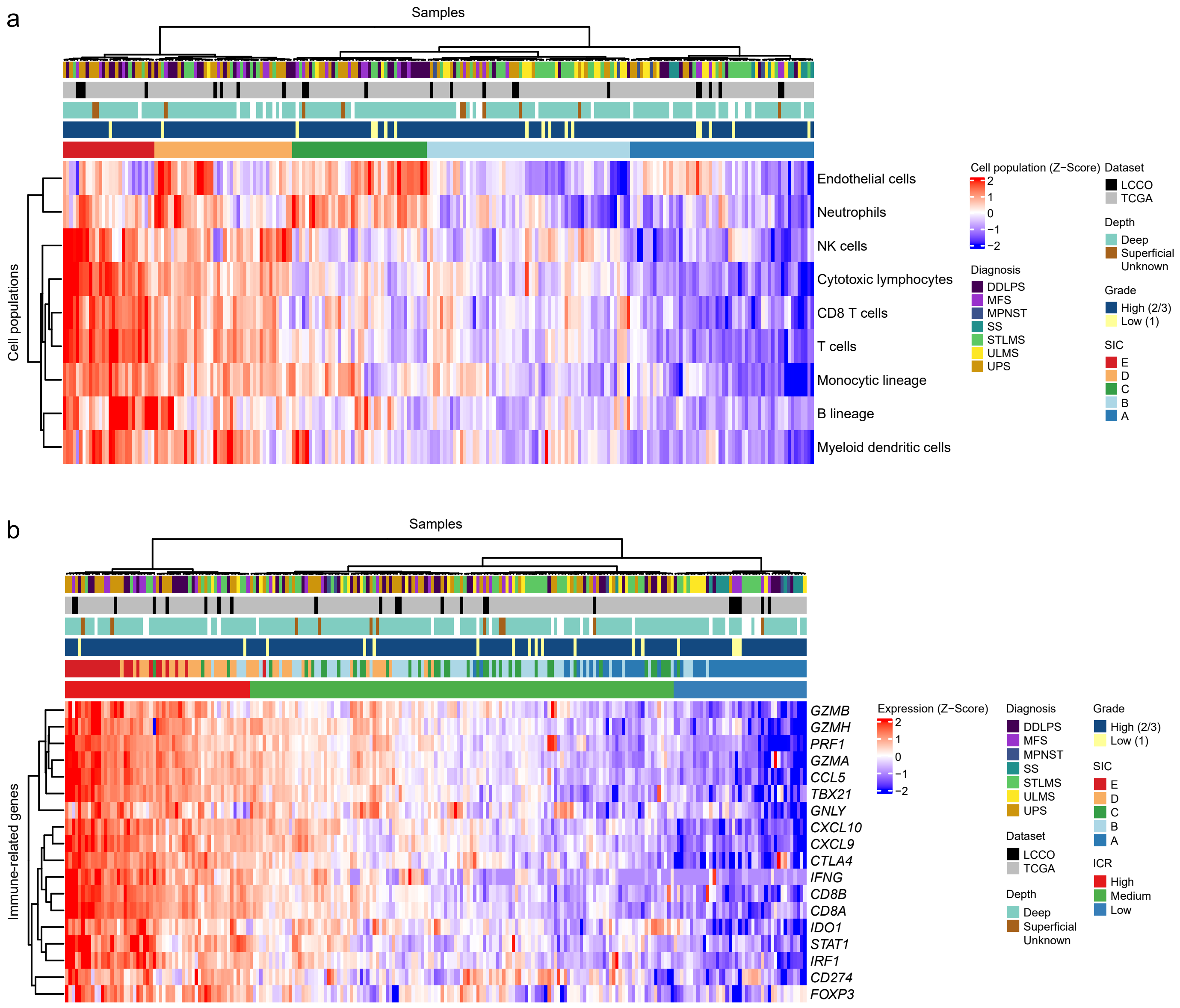
a
Samples
Cell populations
Endothelial cells
Neutrophils
NK cells
Cytotoxic lymphocytes
CD8 T cells
T cells
Monocytic lineage
B lineage
Myeloid dendritic cells
Cell population (Z−Score)
2
1
0
−1
−2
Diagnosis
DDLPS
MFS
MPNST
SS
STLMS
ULMS
UPS
Dataset
LCCO
TCGA
Depth
Deep
Superficial
Unknown
Grade
High (2/3)
Low (1)
SIC
E
D
C
B
A
b
Samples
Immune-related genes
GZMB
GZMH
PRF1
GZMA
CCL5
TBX21
GNLY
CXCL10
CXCL9
CTLA4
IFNG
CD8B
CD8A
IDO1
STAT1
IRF1
CD274
FOXP3
Expression (Z−Score)
2
1
0
−1
−2
Diagnosis
DDLPS
MFS
MPNST
SS
STLMS
ULMS
UPS
Dataset
LCCO
TCGA
Depth
Deep
Superficial
Unknown
Grade
High (2/3)
Low (1)
SIC
E
D
C
B
A
ICR
High
Medium
Low

Supplementary Fig. 2 Clustering using the SIC and ICR signatures in UPS and MFS. **a)** Heatmap presenting the estimated cell population z-scores of the microenvironment cell population (MCP) counter in the LCCO and TCGA cohorts, resulting in five SIC phenotypes. Samples are annotated for diagnosis, dataset, tumor depth, tumor grade and SIC classification. **b)** Heatmap presenting the gene expression z-scores of 18 ICR-genes in the LCCO and TCGA cohorts, resulting in three ICR categories. Samples are annotated for diagnosis, dataset, tumor depth, tumor grade, SIC classification and ICR category. Abbreviations: DDLPS = dedifferentiated liposarcoma; MPNST = malignant peripheral nerve sheath tumor; SS = synovial sarcoma; STLMS = soft tissue leiomyosarcoma; ULMS = uterine leiomyosarcoma

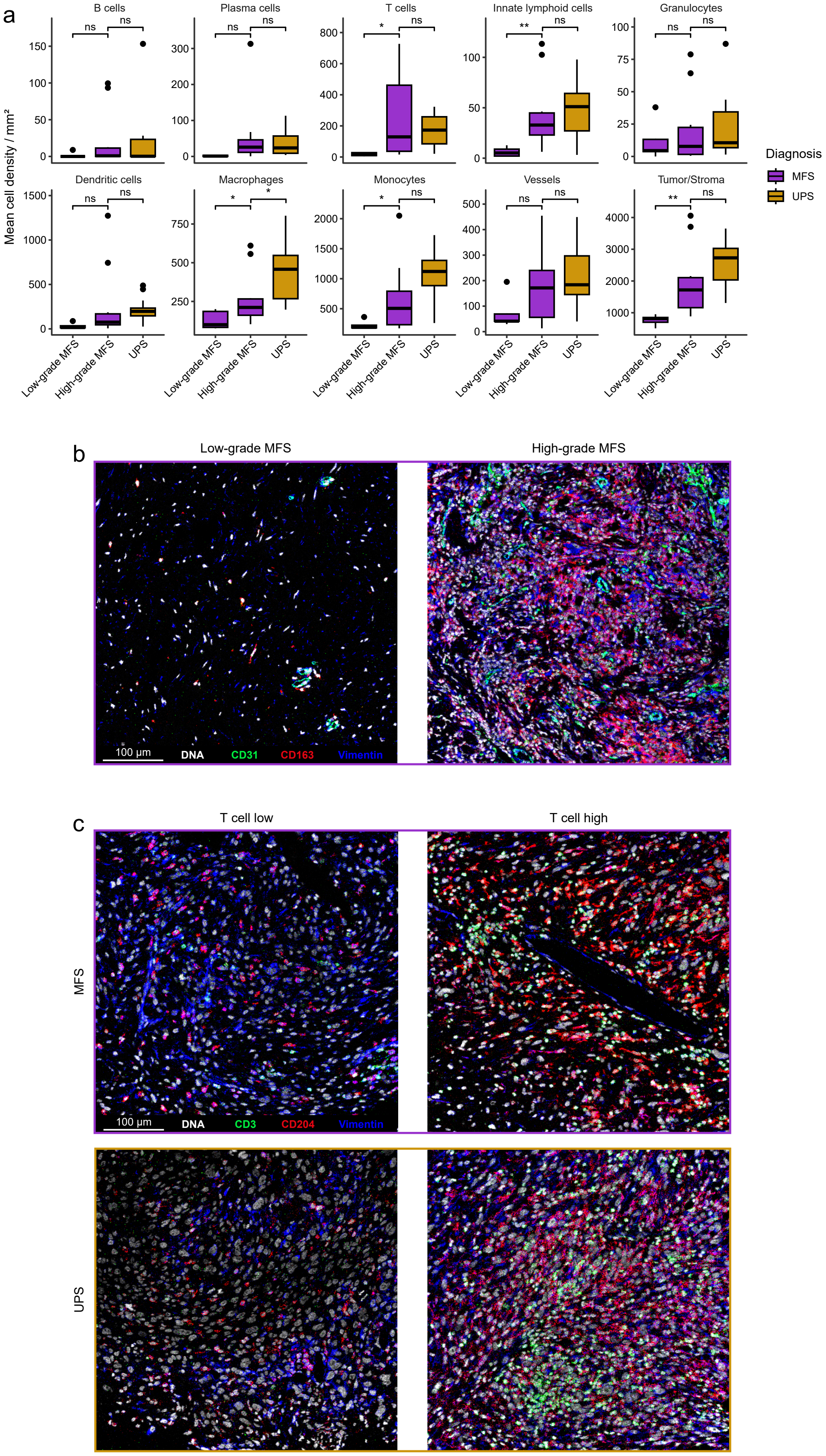
a
Mean cell density / mm²
B cells
Plasma cells
T cells
Innate lymphoid cells
Granulocytes
Dendritic cells
Macrophages
Monocytes
Vessels
Tumor/Stroma
ns
*
**
Low-grade MFS
High-grade MFS
UPS
Diagnosis
MFS
UPS
b
Low-grade MFS
High-grade MFS
100 µm
DNA
CD31
CD163
Vimentin
c
T cell low
T cell high
MFS
UPS
100 µm
DNA
CD3
CD204
Vimentin

Supplementary Fig. 3 Overview of immune contextures in UPS and MFS. **a)** Boxplots presenting the mean cell density per mm^2 of major cell types for low-grade MFS, high-grade MFS and UPS. Differences between low-grade and high-grade MFS, as well as high-grade MFS and UPS were evaluated with a student's t-test. ns = not significant, * = $P < 0.05$, ** = $P < 0.01$. **b)** Example IMC images of a low-grade and a high-grade MFS, highlighting the difference in cellularity. The images display tumor/stromal cells (vimentin in blue), vessels (CD31 in green) and myeloid cells (CD163 in red). **c)** Example IMC images of a T cell low and a T cell high, high-grade MFS (top row) and UPS (bottom row). The images display T cells (CD3 in green), tumor/stromal cells (vimentin in blue) and myeloid cells (CD204 in red)

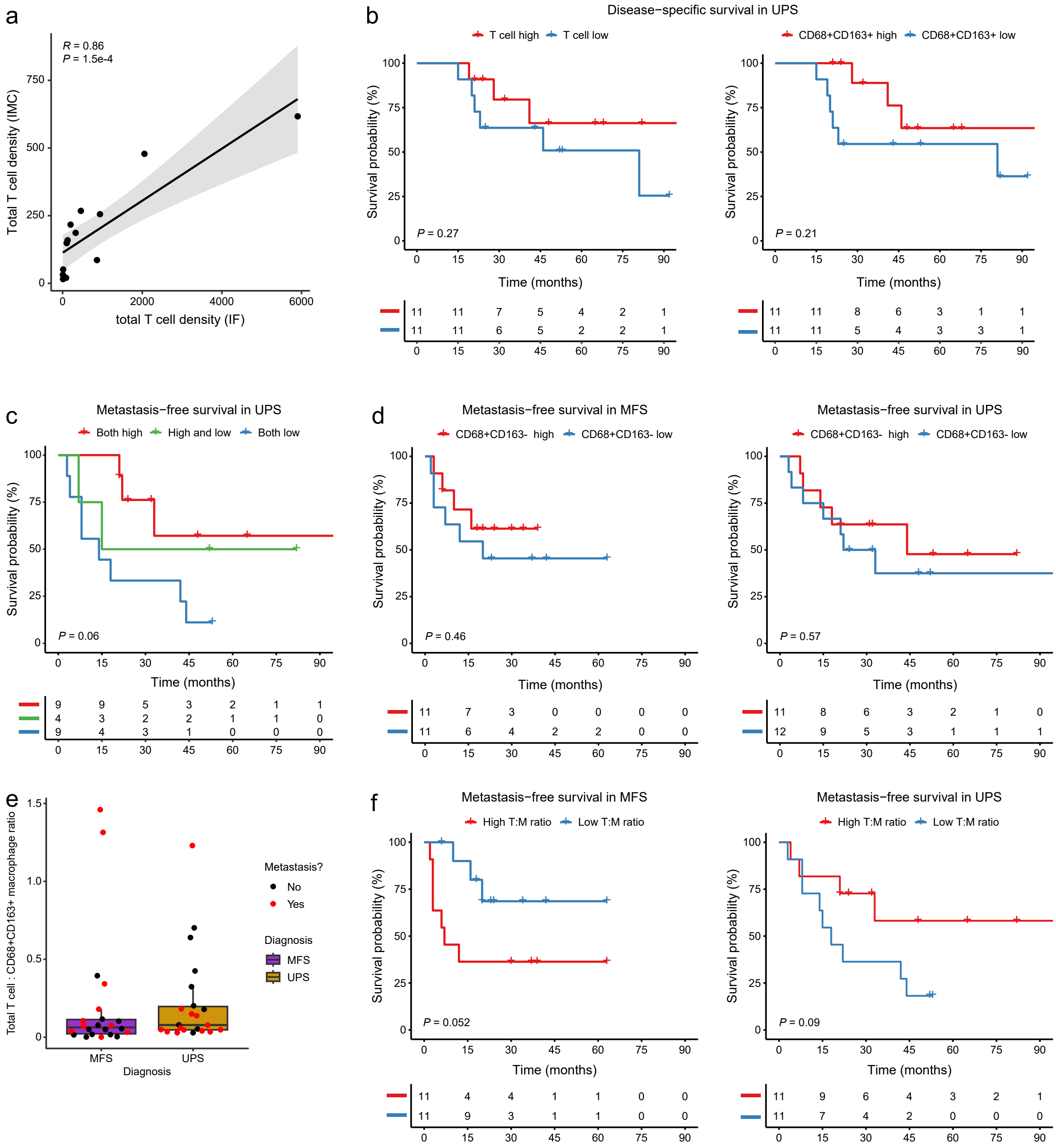

a
R = 0.86
P = 1.5e-4
Total T cell density (IMC)
total T cell density (IF)
b
Disease−specific survival in UPS
T cell high
T cell low
CD68+CD163+ high
CD68+CD163+ low
Survival probability (%)
Time (months)
P = 0.27
P = 0.21
c
Metastasis−free survival in UPS
Both high
High and low
Both low
P = 0.06
d
Metastasis−free survival in MFS
Metastasis−free survival in UPS
CD68+CD163- high
CD68+CD163- low
P = 0.46
P = 0.57
e
Total T cell : CD68+CD163+ macrophage ratio
Metastasis?
No
Yes
Diagnosis
MFS
UPS
f
High T:M ratio
Low T:M ratio
P = 0.052
P = 0.09

Supplementary Fig. 4 Association between immune infiltration and survival in UPS and MFS. **a)** Correlation plot presenting the positive correlation between T cell densities as detected with IMC compared to IF. **b)** Survival analysis of the disease-specific survival in UPS, grouped based on the median total T cell and median $CD68^+CD163^+$ macrophage infiltration. **c)** Survival analysis of the metastasis-free survival in UPS, grouped based on the combined total T cell and $CD68^+CD163^+$ macrophage infiltration. **d)** Survival analysis of the metastasis-free survival in UPS and MFS, grouped based on the median $CD68^+CD163^-$ macrophage infiltration. **e)** Boxplots displaying the T cell-to-$CD68^+CD163^+$ macrophage ratio, separated per subtype. Samples are colored red when that patient has experienced metastatic disease. **f)** Survival analysis of the metastasis-free survival in UPS and MFS, grouped based on the median T cell-to-$CD68^+CD163^+$ macrophage (T:M) ratio. The significance of the Kaplan-Meier curves is presented by the log-rank *P* values

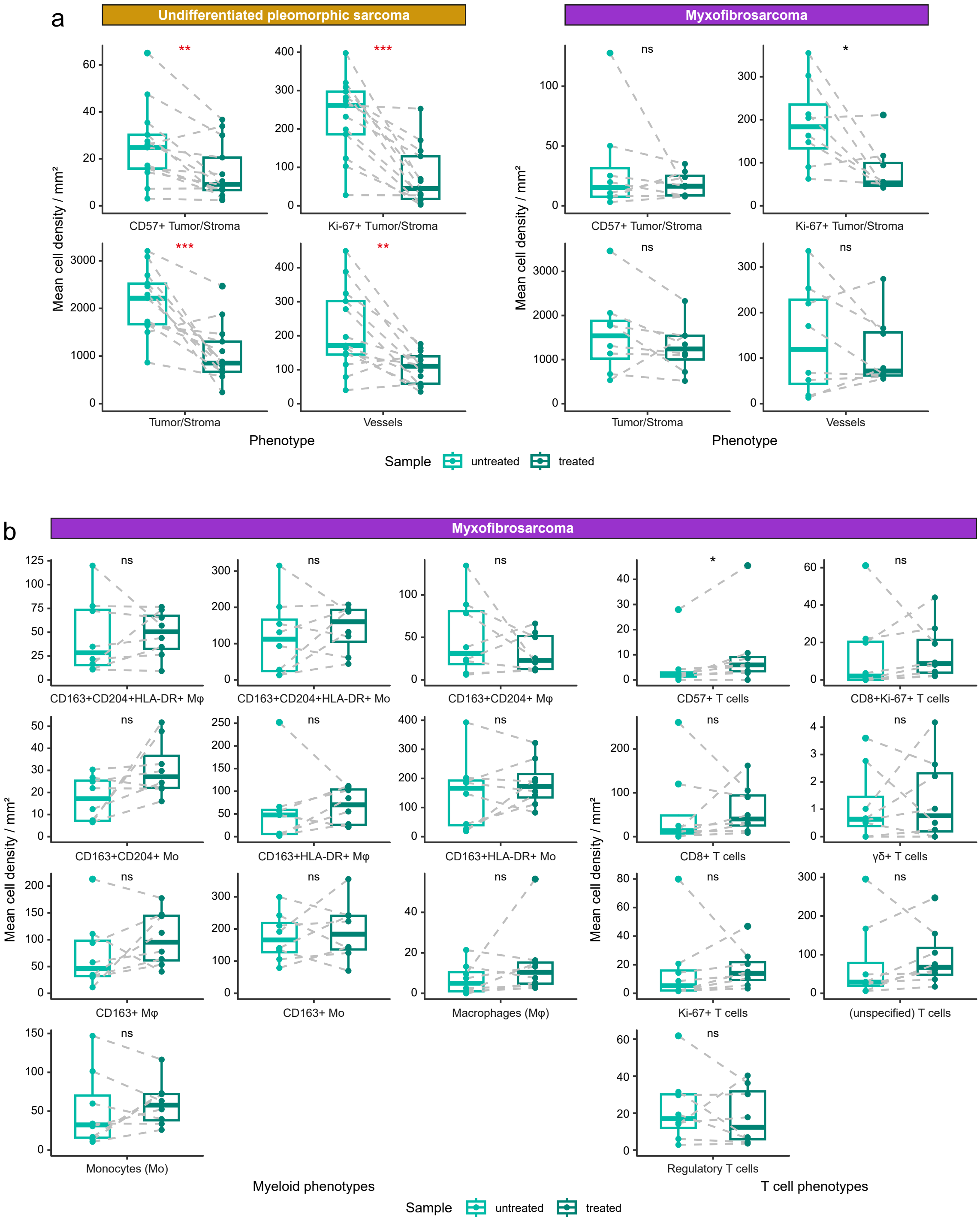

a
Undifferentiated pleomorphic sarcoma
Myxofibrosarcoma
Mean cell density / mm²
CD57+ Tumor/Stroma
Ki-67+ Tumor/Stroma
Tumor/Stroma
Vessels
**
***
***
**
ns
*
ns
ns
Phenotype
Phenotype
Sample
untreated
treated
b
Myxofibrosarcoma
Mean cell density / mm²
CD163+CD204+HLA-DR+ Mφ
CD163+CD204+HLA-DR+ Mo
CD163+CD204+ Mφ
CD163+CD204+ Mo
CD163+HLA-DR+ Mφ
CD163+HLA-DR+ Mo
CD163+ Mφ
CD163+ Mo
Macrophages (Mφ)
Monocytes (Mo)
CD57+ T cells
CD8+Ki-67+ T cells
CD8+ T cells
γδ+ T cells
Ki-67+ T cells
(unspecified) T cells
Regulatory T cells
Myeloid phenotypes
T cell phenotypes
Sample
untreated
treated

Supplementary Fig. 5 The effect of radiotherapy on UPS and MFS. **a)** Paired boxplots presenting the other statistically significant alterations to the UPS immune microenvironment after radiotherapy. The same phenotypes are shown for MFS as a means of comparison. The significance level was evaluated with a student's t-test followed by a Benjamini-Hochberg false discovery rate (FDR) correction. The significance is indicated per phenotype and FDR-significant phenotypes are indicated in red. ns = not significant, * = $P < 0.05$, ** = $P < 0.01$, *** = $P < 0.001$. **b)** Comparison of pre- vs post-treatment immune cell phenotypes in MFS, presented in paired boxplots. The same phenotypes are presented in **Fig. 4** for UPS. The significance is indicated per phenotype and FDR-significant phenotypes are indicated in red. Abbreviations: Mϕ = macrophages; Mo = monocytes

Supplementary table 1. IMC marker panel. Ab = antibody, ON = overnight, PDPN = podoplanin.

	Target	Clone	Metal	Incubation Time	Temp	Dilution (x)
Lymphoid	CD103	EPR4166(2)	168 Er	5h	RT	50
	CD19	D4V4B	172 Yb	5h	RT	100
	CD20	H1	142 Nd	Overnight	4C	100
	CD27	EPR8569	175 Lu	Overnight	4C	50
	CD3	EP449E	153 Eu	Overnight	4C	50
	CD38	EPR4106	169 Tm	Overnight	4C	100
	CD4 + 2nd AB	EPR6855	145 Nd	Indirect ON	4C	100
	CD7	EPR4242	174 Yb	5h	RT	100
	CD8a	D8A8Y	146 Nd	5h	RT	50
	FOXP3	D608R	159 Tb	Overnight	4C	50
	TCRγδ + 2nd Ab	H41	148 Nd	Indirect ON	4C	50
Myeloid	CD11b	D6X1N	144 Nd	5h	RT	100
	CD11c	EP1347Y	176 Yb	5h	RT	100
	CD14	D7A2T	163 Dy	5h	RT	100
	CD15	MC480	171 Yb	Overnight	4C	100
	CD163	D6U1J	173 Yb	5h	RT	50
	CD204	J5HTR3	164 Dy	5h	RT	50
	CD68	D4B9C	143 Nd	Overnight	4C	100
	HLA-DR	TAL 1B5	141 Pr	5h	RT	100
Tumor/Stroma	CD31	89C2	147 Sm	Overnight	4C	100
	CD39	EPR20627	157 Gd	5h	RT	100
	CD45	D9M8I	149 Sm	Overnight	4C	50
	CD45RO	UCHL1	165 Ho	Overnight	4C	100
	CD56	E7X9M	167 Er	5h	RT	100
	CD57	HNK-1 / Leu-7	151 Eu	Overnight	4C	100
	D2-40 (PDPN)	D2-40	166 Er	Overnight	4C	100
	Keratin	C11 and AE1/AE3	198 Pt	Overnight	4C	50
	TGF-β	TB21	115 In	5h	RT	100
	Vimentin	D21H3	194 Pt	Overnight	4C	50
	β-Catenin	D10A8	89 Y	Overnight	4C	100
Activation	Granzyme B	D6E9W	150 Nd	5h	RT	100
	ICOS	D1K2T(TM)	161 Dy	5h	RT	50
	IDO	D5J4E(TM)	162 Dy	Overnight	4C	100
	Ki-67	8D5	152 Sm	Overnight	4C	100
	LAG-3	D2G40(TM)	155 Gd	5h	RT	50
	PD-1	D4W2J	160 Gd	5h	RT	50
	PD-L1	E1L3N(R)	156 Gd	Overnight	4C	50
	Tbet	4B10	170 Er	5h	RT	50
	TIM-3	D5D5R(TM)	154 Sm	5h	RT	100
	VISTA	D1L2G(TM)	158 Gd	5h	RT	100
DNA	Histone H3	D1H2	209 Bi	Overnight	4C	50

Supplementary Table 2. Cell types and used lineage markers for the IMC analysis.

**Phenotype**	**Cell type**	**Lineage markers**
B cells	B cells	CD20+
Plasma cells	Plasma cells/Plasmablasts	CD38+
HLA-DR+<br>CD11c+HLA-DR+	Dendritic cells	CD14-CD68-
Granulocytes	Granulocytes	CD15+
CD56+<br>CD57+<br>Innate lymphoid cells<br>Ki-67+	Innate lymphoid cells	CD3-CD7+
CD163+CD204+<br>CD163+HLA-DR+<br>CD163+<br>CD163+CD204+HLA-DR+<br>Macrophages	Macrophages	CD68+
CD163+CD204+HLA-DR+<br>CD163+CD204+<br>CD163+HLA-DR+<br>CD163+<br>Monocytes	Monocytes	CD14+CD68-
CD57+<br>CD8+Ki-67+<br>CD8+<br>γδ+<br>Ki-67+<br>T cells<br>Regulatory T cells	T cells	CD3+
CD56+<br>CD57+<br>PDPN+<br>Ki-67+<br>Tumor/Stroma	Tumor/Stroma	Vimentin+lineage markers-
Vessels	Vessels	CD31+

Supplementary Table 3. Univariate cox proportional hazard results for disease-specific and metastasis-free survival in UPS. The survival analysis includes 30 UPS patients who were neoadjuvantly treated with radiotherapy. Surgical margin was excluded because all UPS patients had R0 resection margins. Pathologic response was considered <5% vital tumor after treatment. Abbreviations: CI = confidence interval; HR = hazard ratio.

UPS		
**Disease-specific survival**	**Univariate**	
Variable	**HR (95% CI)**	**log-rank *P***
**Age at diagnosis**	1.01 (0.98 - 1.06)	0.7
**Sex (Female)**		
Male	0.62 (0.19 - 2.03)	0.4
**Location (Lower extremities)**		
Other[a]	1.3e-08 (0 - Inf)	0.3
Upper extremities	2.1 (0.43 - 10)	
**Max tumor size (cm)[b]**	1.05 (0.96 - 1.2)	0.3
**Pathologic response (Good)**		
Poor	0.46 (0.14 - 1.5)	0.2

a: Other includes tumors from the trunk and the head & neck area.
B: Tumor size was missing for one tumor from the head & neck area.

UPS		
**Metastasis-free survival**	**Univariate**	
Variable	**HR (95% CI)**	**log-rank *P***
**Age at diagnosis**	1.02 (0.98 - 1.06)	0.3
**Sex (Female)**		
Male	0.62 (0.24 - 1.6)	0.3
**Location (Lower extremities)**		
Other	1.3e-08 (0 - Inf)	0.06
Upper extremities	2.8 (0.88 - 8.7)	
**Max tumor size (cm)**	1.03 (0.95 - 1.1)	0.4
**Pathologic response (Good)**		
Poor	0.79 (0.31 - 2)	0.6

Supplementary table 4. Univariate and multivariate cox proportional hazard results for disease-specific and metastasis-free survival in MFS. The survival analysis includes 24 high-grade MFS patients who were neoadjuvantly treated with radiotherapy. Pathologic response was considered <5% vital tumor after treatment. Abbreviations: CI = confidence interval; HR = hazard ratio.

Myxofibrosarcoma				
**Disease-specific survival**	**Univariate**		**Multivariate**	
Variable	**HR (95% CI)**	**log-rank *P***	**HR (95% CI)**	**log-rank *P***
**Age at diagnosis**	1.1 (0.98 - 1.3)	0.1		
**Sex (Female)**				
Male	1.4 (0.26 - 7.7)	0.7		
**Margin (R0)**				
R1/R2	1.3 (0.15 - 12)	0.8		
**Location (Lower extremities)**				
Trunk	0.58 (0.066 - 5.1)	0.4		
Upper extremities	3.3 (0 - Inf)			
**Tumor depth (Deep)**				
Superficial	7.1e-10 (0 - Inf)	**0.03 ***	2.4e-09 (0 - Inf)	**0.002 ****
**Max tumor size**	**1.3 (1.1 - 1.5) ****	**4e-04 *****	1.2 (0.97 - 1.4)	
**Pathologic response (Good)**				
Poor	**0.096 (0.015 - 0.6) ***	**0.002 ****	0.57 (0.054 - 6)	

Myxofibrosarcoma				
**Metastasis-free survival**	**Univariate**		**Multivariate**	
Variable	**HR (95% CI)**	**log-rank *P***	**HR (95% CI)**	**log-rank *P***
**Age at diagnosis**	1 (0.96 - 1.1)	0.4		
**Sex (Female)**				
Male	1.8 (0.45 - 6.9)	0.4		
**Location (Lower extremities)**				
Trunk	0.45 (0.56 - 3.6)	0.6		
Upper extremities	0.45 (0.56 - 3.6)			
**Margin (R0)**				
R1/R2	0.79 (0.1 - 6.3)	0.8		
**Tumor depth (Deep)**				
Superficial	0.14 (0.17 - 1.08)	**0.03 ***	0.22 (0.026 - 1.9)	**0.005 ****
**Max tumor size (cm)**	**1.2 (1.04 - 1.3) ****	**0.004 ****	1.1 (0.97 - 1.2)	
**Pathologic response (Good)**				
Poor	**0.17 (0.044 - 0.66) ***	**0.004 ****	0.46 (0.087 - 2.4)	
